# Trade-Offs between the Metabolic Rate and Population Density of Plants

**DOI:** 10.1371/journal.pone.0001799

**Published:** 2008-03-19

**Authors:** Jian-Ming Deng, Tao Li, Gen-Xuan Wang, Jing Liu, Ze-Long Yu, Chang-Ming Zhao, Ming-Fei Ji, Qiang Zhang, Jian-quan Liu

**Affiliations:** 1 MOE Key Laboratory of Arid and Grassland, School of Life Science, Lanzhou University, Lanzhou, China; 2 Institute of Agroecology and Ecoengineering, College of Life Sciences, Zhejiang University, Hangzhou, China; 3 Institute of Modern Physics, Chinese Academy of Sciences, Lanzhou, Gansu; Centre National de la Recherche Scientifique, France

## Abstract

The energetic equivalence rule, which is based on a combination of metabolic theory and the self-thinning rule, is one of the fundamental laws of nature. However, there is a progressively increasing body of evidence that scaling relationships of metabolic rate *vs*. body mass and population density *vs*. body mass are variable and deviate from their respective theoretical values of 3/4 and −3/4 or −2/3. These findings questioned the previous hypotheses of energetic equivalence rule in plants. Here we examined the allometric relationships between photosynthetic mass (*M*_p_) or leaf mass (*M*_L_) *vs.* body mass (*β*); population density *vs.* body mass (*δ*); and leaf mass *vs*. population density, for desert shrubs, trees, and herbaceous plants, respectively. As expected, the allometric relationships for both photosynthetic mass (i.e. metabolic rate) and population density varied with the environmental conditions. However, the ratio between the two exponents was −1 (i.e. *β*/*δ* = −1) and followed the trade-off principle when local resources were limited. Our results demonstrate for the first time that the energetic equivalence rule of plants is based on trade-offs between the variable metabolic rate and population density rather than their constant allometric exponents.

## Introduction

Many studies of mammals suggested that the relationship between basal metabolic rate and body mass can be expressed as the 3/4 power of the former [1]–[3]. In addition, when analyzing mammalian data from a wide variety of habitats across the world, Damuth [4]–5 showed that population density was inversely scaled with body size and had an allometric exponent of −3/4. By combining both scaling relationships, they further proposed the energetic equivalence rule, which states that the amount of energy per unit area used by a population of a specific species is independent of body size. Recently, West et al. [6]–[8] developed a general mechanistic model (the WBE model), based on the fractal volume-filling theory, to predict and explain the 3/4 scaling exponent for animals and plants. Enqusit et al. [9] extended the energetic equivalence rule from mammal populations to plant populations based on the WBE theory, *R* = *N*_max_ *Q*∝*M* ^3/4^ *M* ^−3/4^ = *M* ^0^, where *R* is the rate of resource use per unit area; *N*_max_ is the maximum population density; *Q* is the average rate of resource use or the metabolic rate per individual; and *M* is the average individual mass. These authors then extrapolated their data and concluded that the allometric exponent of the density–mass relationship for plants should be −4/3 rather than −3/2, and that energy-equivalence as a general model could be applicable to all plant populations in any environment [9]–[12].

The researchers who developed the WBE theory have claimed that the 3/4 exponent contains mathematical errors and is derived on the basis of an explicit assumption [13]–[16] and therefore does not generally apply to all organisms. Recent analyses of very large data sets on the basal metabolic rates of mammals and birds support a 2/3 exponent, rather than 3/4, derived from Eucidean geometric scaling [13], [17]. In addition, the −3/2 power rule for plants (i.e. *N∝M^−^* ^2/3^) was ever treated as a general principle of plant population biology [18]–[19] and the total energy or resource use per unit area for a population can be expressed as: *R* = *N*_max_ *Q*∝*M* ^2/3^ *M* ^−2/3^ = *M* ^0^ = constant. Both of these models show that the rate of resource use per unit area is independent of plant size, although both models assume different allometric exponents. It remains unclear whether the energy equivalence relationship can be derived from *R*∝*M* ^3/4^ *M* ^−3/4^ *= M* ^0^ or *R*∝*M* ^2/3^ *M* ^−2/3^∝*M* ^0^.

The process for examining the applicability of the energy equivalence model is difficult and appears logically inappropriate (for discussion see [9]). In our study, we assumed that the relationship between the rate of limiting resource use per unit area and the mean plant size can be described by *R* = *KM^a^* (where *K* is a constant and α is an exponent). Since the rate of resource supply per unit area is limiting, the dependent variable *R* is a constant *K ′* so that the exponent (α) of the independent variable *M* must be zero for any body size, as *R* = *KM* ^0^ = *K ′*. Therefore, the energy equivalence rule *R*∝*M* ^0^ should be suitable for any given environment only if the population resource is limited [20]. The process of examining the applicability of the energy equivalence rule is mathematically difficult, and the −4/3 power rule derived from this energetic equivalence rule has been criticized and questioned by several authors [20]–[22]. In fact, many authors have suggested that the allometric exponents for the metabolic rate can vary with some biotic and abiotic factors [15], [23]–[26], just as the slopes of self-thinning lines vary between species, shade tolerance and site quality [20], [22], [27]–[31]. If the allometric exponent of the average rate of resource use per individual *Q* vs. plant mass is *β*, i.e. *Q*∝*M^β^* (where *β* is variable), according to the general model of energy equivalence, *R* = *N*_max_ *Q*∝*M* ^0^, the relationship between population density and plant mass should theoretically follow the model: *N*_max_∝*M^δ^*, where *δ* = -*β* or *δ/β* = −1. Currently, it is unclear if this trade-off relationship between the two scaling exponents is valid in natural systems. It is, therefore, necessary to investigate any synchrony in the scaling relationships between metabolic rate and population density, and plant body mass, under different environmental conditions and for different plant types. We will also examine the variability in the allometric exponents *β* and *δ* that are 3/4 and −3/4, or 2/3 and −2/3, respectively.

The accurate and consistent measurement of metabolic rates is very difficult [3]. According to the predictions of the WBE model, the metabolic rate of plants, *B*; rate of biomass production, *G*; photosynthetic biomass, *M*_p_ (i.e. the total leaf biomass, *M*_L_); covary and all should be proportional to the 3/4 power of total plant mass, *M*, i.e. *B*∝*G*≈*M_P_*(*orM_L_*)∝M^3/4^ [3], [6]–[8]. These scaling relationships have been demonstrated by a large number of authors, especially for angiosperms and gymnosperms [32]–[36]. The rate of biomass production, however, may not adequately reflect the metabolic rate or the rate of resource use because growth rates only provide estimates of an organism's net assimilation, and exclude any dissimilation energy. For the energetic equivalence rule, the average resource use rate or metabolic rate of individual plants, *Q*, should be more appropriately replaced by photosynthetic biomass, *M_L_*. Assuming that the rate of resource use per unit leaf biomass, *K_i_*, is constant in the same plant type (where *i* represents the different plant types or environmental conditions), we can generate the equation: *Q* = *M_L_*×*K_i_*∝*M_L_*. Combining the models: *Q*∝*M^β^*, *N*∝*M^δ^*, and *Q*∝*M_L_*, the relationship between leaf mass and density can be obtained by the equation, *M_L_*∝*N^β/δ^*. If the trade-off relationship between the two scaling exponents holds true, i.e. *δ/β* = −1, the predicted leaf mass–density relationship should be consistent with previous studies [12] that have shown a scaling exponent of −1.

In this study we examined the relationships between metabolic rate and population density, and body size, for a range of plant populations including desert shrubs, different forest types, and monoculture herbages. Our analysis of these data, which spans a size range of 11 orders of magnitude, unequivocally shows that allometric relationships between photosynthetic mass (*M*_p_) or leaf mass (*M*_L_), and population density and body mass varied greatly between desert shrubs, trees, and herbaceous plants, respectively. We further confirmed that the energetic equivalence rule of plants is based on trade-offs between the variable metabolic rate and population density.

## Results

**Figure 1:**
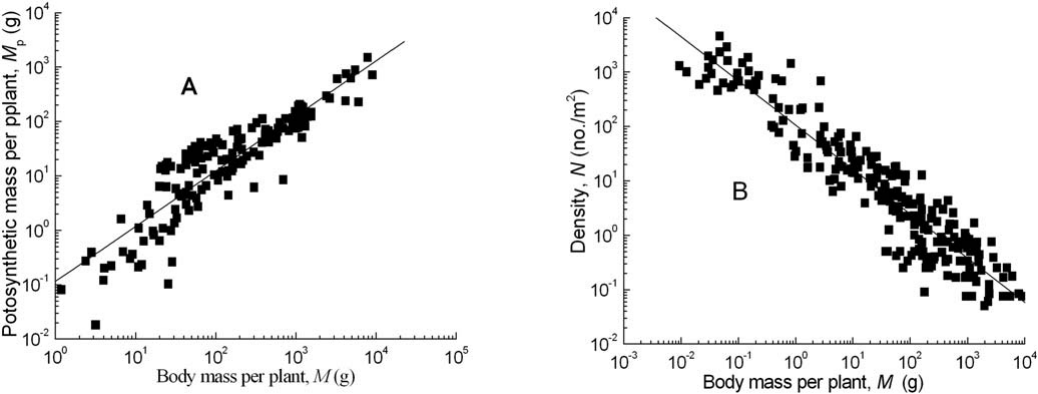
The allometric relationships between photosynthetic mass and body mass (A), and population density and body mass (B) for desert shrubs. All regressions are significant at *P*<0.0001 and the 95% CI of the slopes are statistically different from 3/4 and −3/4 (also see Table 1, 2), but the ratio of the two exponents is not statistically different from −1.

**Figure 2:**
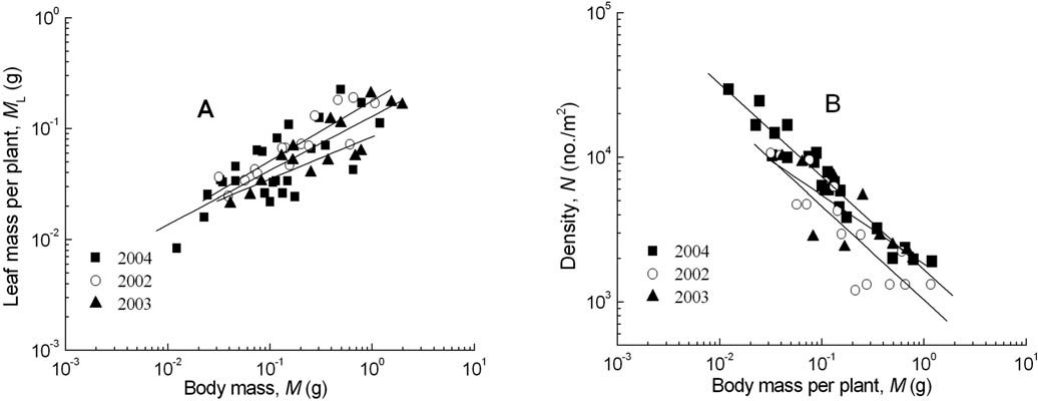
The allometric relationships between photosynthetic mass and body mass (A), and population density and body mass (B) for dense spring wheat populations. All regressions are significant at *P*<0.0001 and the 95% CI of the slopes are statistically different from 3/4 and −3/4 (also see Table 1, 2), but the ratio of the two exponents is not statistically different from −1.

The scaling exponents (*β*) of photosynthetic mass and body mass were statistically analyzed and ranged between 0.47 and 1.06 for the trees, shrubs and herbaceous plants (Table 1). Among the 17 forest types, the 95% confidence intervals (CIs) showed that only one scaling exponent included 3/4, three values contained both 2/3 and 3/4, three values included 2/3, and the ten remaining values fell outside the 2/3 and 3/4 range. For desert shrubs and spring wheat, the CIs were above 3/4 and below 2/3, respectively (Fig. 1A, Fig. 2A, Table 1).

**Table 1:** The scaling relationship between photosynthetic biomass, *M*_p_, and the body mass, *M*, for trees, desert shrubs and herbaceous plants.

Plant type	*n*	Slope, *β*±SE	IT	95% CI	*r* ^2^
**Trees/Forest**					
Boreal/temperate ***Larix*** forest	48	0.97±0.017	−1.35	0.93, 1.00	0.986
Boreal/alpine ***Picea abies*** forest	168	0.61±0.023	0.81	0.53, 0.67	0.761
Boreal ***Pinus sylvestris*** var. ***mongolica*** forest	10	0.65±0.040	0.44	0.56, 0.69	0.970
Temperate ***Pinus tabulaeformis*** forest	154	0.87±0.023	−0.45	0.82, 0.93	0.897
Temperate mixed coniferous-broadleaved forest	22	0.57±0.037	0.88	0.51, 0.59	0.915
Temperate typical deciduous broadleaved forest	165	1.06±0.041	−1.51	0.94, 1.20	0.758
Temperate/subtropical montane ***Populus-Betula*** deciduous forest	127	0.99±0.026	−1.39	0.93, 1.05	0.911
Desert riverside woodland	9	0.82±0.14	−0.84	0.66, 0.90	0.791
Subtropical mixed evergreen-deciduous broadleaved forest	22	0.89±0.079	−0.81	0.79, 1.22	0.841
Subtropical evergreen broadleaved forest	238	0.94±0.015	−1.08	0.92, 0.96	0.940
Sclerophyllous evergreen ***Quercus*** forest	9	0.87±0.049	−0.76	0.81, 0.93	0.977
Tropical rainforest and monsoon forest	13	0.96±0.13	−1.14	0.35, 1.12	0.793
Subtropical montane ***Pinus yunnanensis*** and ***P. khasya*** forest	46	0.93±0.02	−0.88	0.89, 0.96	0.980
Subtropical ***Pinus massoniana*** forest	66	0.92±0.052	−0.84	0.83, 1.02	0.798
Subtropical montane ***Pinus armandii***, ***P. taiwanensis*** and ***P. densada***	55	0.77±0.034	−0.013	0.71, 0.85	0.899
Subtropical ***Cunninghamia lanceolata*** forest	98	0.81±0.065	−0.21	0.65, 0.97	0.382
Subtropical montane ***Cupressus*** and ***Sabina*** forest	16	0.55±0.084	1.14	0.43, 0.67	0.677
**Shrubs**					
Desert shrubs	148	0.92±0.047	−0.71	0.84, 1.00	0.618
**Herbages**					
Spring wheat 2002	15	0.61±0.075	−0.70	0.48, 0.70	0.803
Spring wheat 2003	10	0.47±0.096	−1.02	0.36, 0.69	0.662
Spring wheat 2004	23	0.65±0.097	−0.75	0.50, 0.72	0.528

SE is the standard error, and CI is the confidence interval of the slope.

The regression slopes of population density *vs*. body mass among the forest types had high variability and ranged from −0.52 to −1.15. Among the 17 forest types, two slope values had 95% CIs that contained 2/3, three contained 3/4, six had both 2/3 and 3/4 within them, and six fell outside the 2/3 and 3/4 range (Table 2). The CI for the desert shrubs was greater than the theoretical value of 3/4 (Fig. 1B). The regression slope for spring wheat in 2002 was very close to −3/4 (Fig. 2B). These slopes were close to −2/3 in 2003 and 2004, although they were significantly different from 3/4 (Table 2).

**Table 2:** The scaling relationship between population density, *N*, and body mass, *M*, and the ratio of the allometric exponent *δ* to *β* for trees, desert shrubs and herbaceous plants.

Plant type	*n*	Slope, *δ*±SE	IT	95% CI	*r* ^2^	*δ/β*
**Trees/Forest**						
Boreal/temperate ***Larix*** forest	48	−0.81±0.040	3.20	−0.88, −0.74	0.887	−0.84
Boreal/alpine ***Picea abies*** forest	168	−0.671±0.023	2.50	−0.72, −0.61	0.802	−1.10
Boreal ***Pinus sylvestris*** var. ***mongolica*** forest	10	−0.70±0.055	2.51	−0.76, −0.51	0.952	−1.08
Temperate ***Pinus tabulaeformis*** forest	154	−0.81±0.037	2.88	−0.90, −0.71	0.691	−0.93
Temperate mixed coniferous-broadleaved forest	22	−0.64±0.027	2.30	−0.67, −0.60	0.964	−1.13
Temperate typical deciduous broadleaved forest	165	−0.88±0.033	3.34	−0.97, −0.81	0.773	−0.83
Temperate/subtropical montane ***Populus-Betula*** deciduous forest	127	−0.89±0.042	3.50	−0.97, −1.82	0.729	−0.90
Desert riverside woodland	9	−1.13±0.15	4.42	−1.44, −1.02	0.877	−1.37
Subtropical mixed evergreen-deciduous broadleaved forest	22	−0.66±0.08	2.48	−0.90, −0.47	0.700	−0.74
Subtropical evergreen broadleaved forest	238	−0.72±0.022	2.85	−0.78, −0.68	0.781	−0.77
Sclerophyllous evergreen ***Quercus*** forest	9	−1.15±0.15	5.21	−1.21, −0.89	0.879	−1.32
Tropical rainforest and monsoon forest	13	−0.76±0.12	3.09	−1.16, −0.64	0.737	−0.79
Subtropical montane ***Pinus yunnanensis*** and ***P. khasya*** forest	46	−0.85±0.033	3.31	−0.91, −0.77	0.933	−0.90
Subtropical ***Pinus massoniana*** forest	66	−0.73±0.051	2.74	−0.84, −0.63	0.685	−0.79
Subtropical montane ***Pinus armandii***, ***P. taiwanensis*** and ***P. densada***	55	−0.71±0.038	2.59	−0.79, −0.66	0.844	−0.92
Subtropical ***Cunninghamia lanceolata*** forest	98	−0.52±0.047	1.78	−0.64, −0.44	0.211	−0.64
Subtropical montane ***Cupressus*** and ***Sabina*** forest	16	−0.66±0.091	2.40	−0.84, −0.55	0.731	−1.19
**Shrubs**						
Desert shrubs	239	−0.87±0.019	2.10	−0.90, −0.83	0.882	−0.94
**Herbages**						
Spring wheat 2002	15	−0.75±0.10	2.94	−1.08, −0.61	0.752	−1.23
Spring wheat 2003	10	−0.61±0.14	3.17	−0.73, −0.55	0.598	−1.31
Spring wheat 2004	23	−0.66±0.037	3.20	−0.73, −0.62	0.936	−1.03
**Mean value of ** ***β/δ*** **±SE**					**−0.99**±0.21	

SE is the standard error, and CI is the confidence interval of the slope.

**Figure 3:**
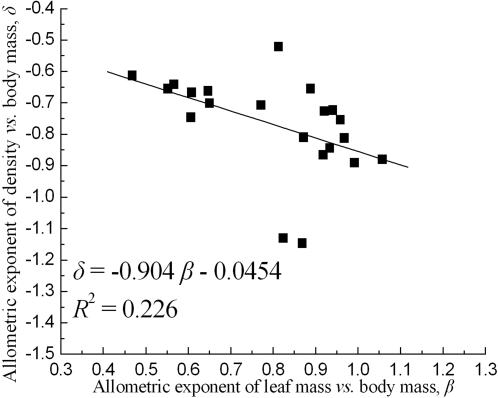
The regression relationships between the two allometric exponents for trees, desert shrubs and monoculture herbaceous plants (spring wheat). The slope is very close to −1.

The ratio value, *δ/β*, was calculated for the scaling exponents, *δ* and *β*. The average value of the *δ/β* ratio was −0.99 (Table 2). Furthermore, the regression slope between the two scaling exponents was not statistically different from the predicted value of −1 (Fig. 3).

## Discussion

**Figure 4:**
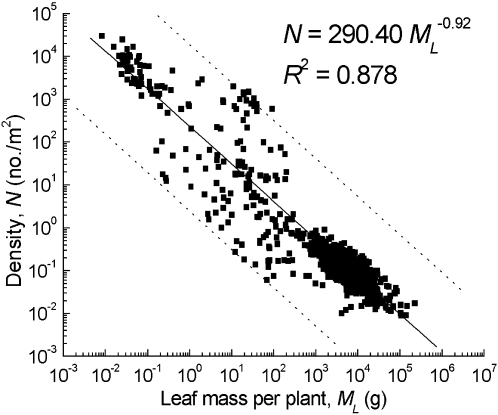
The allometric relationships between population density and photosynthetic mass or leaf mass for trees, desert shrubs and monoculture herbaceous plants (spring wheat). The regression is significant at *P*<0.0001 and the exponent approximates to the theoretical value of −1 (also see Table 1, 2).

Our results showed that the scaling exponents of both photosynthetic mass *vs*. body mass and population density *vs*. body mass varied depending on plant species and habitat type (Table 1, 2). More importantly, the trade-off relationship between the two scaling exponents supported our prediction, i.e. *δ/β* = −1 (Table 2). The regression slope values between the two scaling exponents, and density *vs*. average leaf mass were also close to the theoretical value of −1 (Fig. 3, Fig. 4) and with the previous study by Niklas et al. [12].

There are two main power rules concerning the density/abundance–body size relationship, which are supported by empirical data, i.e. the −3/2 power rule [19], [37]–[40] and the −4/3 power rule [3], [9], [11], [35]–[36], [41]. However, other researchers consider that the allometric exponents vary with environmental factors or between species [20], [22], [27]–[28], [42]–[44]. White [18] suggested that the slopes (i.e. 1/*δ*) of biomass–density relationships varied within the range of −1.8 to −1.3, while Wang and Zhang [45] suggested that the theoretical values of slopes should continuously vary from −1 to -∞ when the growth forms of plants are transformed from a purely horizontal extension to absolute height growth. In fact, according to the predictions of the WBE model, 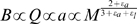 (where 0≤*ε_a_* or *ε_l_*≤1, both of which are arbitrary exponents and *a* is the total plant leaf area; see [7], the theoretical values of exponents should range between 1/2 and 3/4 when plants evolve by natural selection under different conditions of environmental stress [46]. Our results showed that allometric exponents of metabolic rate vary greatly according to a number of biotic and abiotic factors; this supports previous metabolic rate studies [13]–[14], [23]–[26], [47]–[50]. Although there is considerable evidence that the allometric exponents, *β* and *δ*, are variable, we found that these different values follow the trade-off law based on the energetic equivalence rule. Furthermore, this trade-off relationship implies that not only are the two types of exponents variable, but they are also co-dependent.

**Figure 5:**
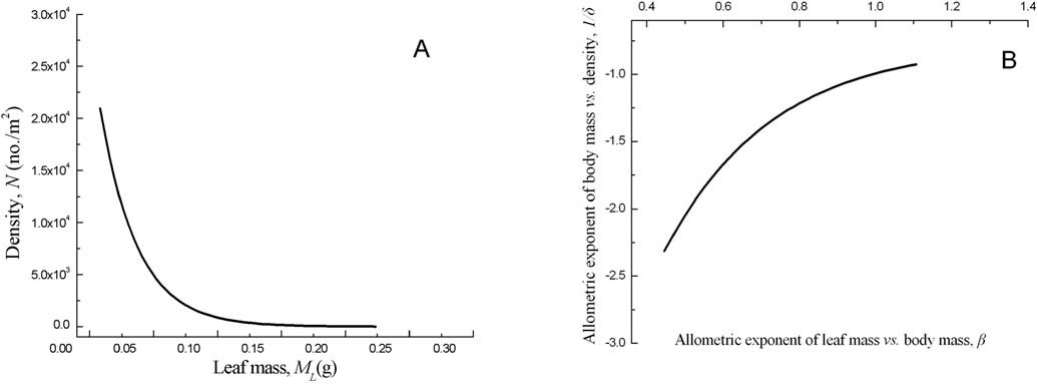
The trade-off relationships between population density *vs*. the photosynthetic mass/leaf mass (A) and the allometric exponents, 1/*δ vs. β* (B). The population density and the photosynthetic mass show a reciprocal relationship (A) and the exponents 1/*δ* and *β* show a negative reciprocal relationship (B).

The trade-off relationship is a fundamental principle of strategy theory in evolutionary ecology [51], which considers that an organism adopts a suitable strategy to survive and grow under a given environment stress. The mechanism of both density–leaf mass and the allometric exponents *δ*, or 1/*δ*-*β* trade-off relationships may derive from intraspecific plant dynamics. Some researchers have suggested that the leaf biomass per individual inversely scales with population density in populations that are undergoing self-thinning [12], [43], [52]. The decline in leaf mass per individual with increasing density results in a decrease in the rate of resource use and the allometric exponent *β* (Fig. 5). Some studies have shown that the exponent, 1/*δ*, depends on the allometric exponent between height and stem diameter [45]–[46]. The latter exponent increases with density because more energy and resources may be allocated to stem height growth as a result of competition [53]–[54]. Thus the dynamics of populations with different plant densities and height–stem diameter relationships largely determine the trade-off relationship between the exponents, *β* and 1/*δ*, especially in dense populations. Moreover, this trade-off relationship indicates that the values of the scaling exponents, *β* and 1/*δ*, should be continuous variables, rather than constants (Fig. 5B). It remains unclear how the scaling exponent between height and stem diameter quantitatively varies with population density or the intensity of competition.

As reported for metabolic rates [55], we found that the leaf mass reflects both photosynthetic ability and the absorbency of water and nutrients from soil. Under drought conditions, the plant individual has the relatively high ratio of photosynthetic mass to body mass may result from the thick leaves and the assimilating shoots of plants and the relatively small body size, which may lead to increases in the photosynthetic efficiency and the capacity to absorb water, and also decrease soil evaporation through canopy shading, thereby enhancing the drought stress resistance [56]–[57]. Moreover, leaves with high ratios of leaf mass/body mass may have further ecological significance in harsh environments; for example, most leaves will fall and decompose to increase soil fertility in nutrient-poor soil [58]. In contrast, high ratios imply that the body size of an individual will be constrained within a relatively small range to reduce the use of the limiting resource and enhance survival ability. The self-thinning phenomenon in plants occurs in dense populations mainly ascribing to the leaf mass per individual (or the value of exponent *β*), which drops sharply with increased density and growth. It is noticeable that the fluctuating scaling relationships of leaf mass and body mass for trees are also dependent on environmental conditions and are species-specific. If only resources are limited, both the leaf mass *vs*. density relationship and the relationship between the scaling exponents, *β* and 1/*δ*, would follow the trade-off law for any given environmental condition. Overall, our results demonstrate for the first time that the energetic equivalence rule of plants is based on trade-offs between the variable metabolic rate and population density rather than their constant allometric exponents.

## Materials and Methods

### Desert shrubs

All of the data used in our analyses were collected in July and August between 2003 and 2007 from shrub-dominated communities at our experimental sites (See the Dataset S2.). The experimental plots are located in the central and western parts of the Gansu province, China (Baiyin, Jingtai, Minqin and Linze sites) between 100°08′ and 104°24′ E longitude and between 39°22′ and 36°29′ N latitude. They are classified as arid and semi-arid regions on the boundaries of the Tengger and Badain Jaran deserts, where the annual mean precipitation ranges between 115 mm and 209 mm. The design of the sampling quadrats and the measurement of the stand density and total biomass for each population are described by Deng et al. [20]. Because the assimilating branches, twigs and petioles (green tissue) of most xerophyte plants have considerable photosynthetic capacity (in addition to the leaves), enabling them to adapt to arid environments, the mass of all the photosynthetic tissues was measured separately for sub-samples of aboveground and belowground parts of each plant species.

### Trees

All of the data used in this study were collected from the primary literature ([59], included in the Dataset S1., also see http://www.geodata.cn). These data spanned a range of latitudes (18°N and 53°N), and altitudes (10 m to 4240 m above sea level), including 1266 plots/populations from six biomes and 17 forest types across China [59]. The species under investigation included angiosperms and gymnosperms.

Luo [59] provided information on the average mass and annual net production for different plant parts (leaf, stem, and root) for different aged plants, densities and species. The units of mass and density were converted from tons of dry matter and the number of plants per ha. to grams per individual plant and number of plants per square meter, respectively.

### Herbage

Field experiments with spring wheat (*Triticum aestivum* L. New Cultivar No.3) were conducted at the Yuzhong experimental station of Lanzhou University, China, from May to July in 2002 and 2004. We also re-analyzed some relevant data from self-thinning experiments of spring wheat populations conducted in 2003 (See the Dataset S3.) [31].

The experimental design combined populations sown at six and ten densities: 1, 10, 100, 1×10^3^, 4×10^3^, and 1×10^4^ seeds m^−2^, and 1, 100, 500, 1×10^3^, 2×10^3^, 6×10^3^, 1×10^4^, 2×10^4^, and 4×10^4^ seeds m^−2^ in 2002 and 2004, respectively, with four replicates for each seed density. The area of each plot was 1 m×1 m, with a 0.3 m-wide buffer zone to avoid any marginal effects. The soil moisture and fertility was sufficient to ensure plant growth without any water or nutrient stress [31]. The stand density, leaf area, leaf biomass and body mass were measured in each plot at the three-leaf, tillering, elongation, heading and ripening stages, respectively. The leaf area was estimated by the Dry Weight Method described by Deng et al. [60]. The mean dry mass data were collected from 50 randomly sampled individuals in the populations with sowing densities >500/m^2^ in four replicate plots at each sowing density. The dry plant mass was weighed after harvesting and being dried by ovens.

### Statistical Analyses

To meet the requirement of the energetic equivalence rule for spring wheat populations, we analyzed the relevant data for closed populations with sowing densities >1000 seeds m^−2^. All the allometric exponents, the intercepts and the 95% confidence intervals were evaluated by Model Type II (reduced major axis i.e. RMA, 1.17version) regression of the log-transformed data. The 95% confidence intervals were used to assess whether an empirically calculated allometric exponent included the predicted theoretical values [32].

## Supporting Information

Dataset S1 The dataset S1 used in the analysis of our paper.
(0.11 MB PDF)

Dataset S2 The dataset S2 was used in the analysis of our paper
(0.13 MB PDF)

Dataset S3 The dataset S3 was used in the analysis of our paper
(0.04 MB PDF)
