# Supplementary material for: Trade-Offs between the Metabolic Rate and Population Density of Plants
Source: PLoS One. 2008 Mar 19;3(3):e1799. doi: 10.1371/journal.pone.0001799 (PMC2265546; doi:10.1371/journal.pone.0001799)
Supplement: Dataset S1 — The dataset S1 used in the analysis of our paper. (0.11 MB PDF) [file pone.0001799.s001.pdf]

Dataset S1. Tree data							
Province/Sites	Alt.(m)	Long.(E,deg.)	Lat.(N,deg.)	Born	L(g/no.)	M(g/no.)	D(no./m^2)
**Boreal/temperate Larix forest**							
Heilongjiang	800	129.40	44.30	natural	17538.0711	610989.8477	0.0394
Heilongjiang	550	124.70	52.30	natural	9312.7148	298384.8797	0.0291
Heilongjiang	441	126.60	51.70	natural	2570.1754	82175.4386	0.1140
Heilongjiang	590	131.80	46.50	natural	13939.3939	422030.3030	0.0330
Heilongjiang	800	130.20	44.10	natural	14375.0000	450643.3824	0.0544
Heilongjiang	590	125.20	51.40	natural	9016.6667	318266.6667	0.0600
Heilongjiang	876	124.20	52.60	natural	2146.9860	50235.3427	0.2422
Heilongjiang	500	128.40	49.50	natural	5896.2963	198770.3704	0.0675
Jilin	880	128.10	42.40	natural	28991.8256	655231.6076	0.0367
Jilin	900	128.27	42.75	natural	14055.5556	683583.3333	0.0360
Jilin	1075	127.30	42.20	natural	48219.1781	1095525.1142	0.0219
Mongolia	650	124.50	52.50	natural	5675.0000	136450.0000	0.0400
Mongolia	700	123.00	50.33	natural	2261.1940	73067.1642	0.1340
Mongolia	800	120.80	52.20	natural	5224.3590	221474.3590	0.0624
Mongolia	800	121.70	50.40	natural	790.9029	25101.8330	0.2946
Mongolia	781	121.50	50.80	natural	6270.0535	222847.5936	0.0748
Mongolia	637	124.00	50.40	natural	6477.8761	223946.9027	0.0565
Mongolia	820	121.40	48.40	natural	530.9370	15162.5737	0.7289
Mongolia	1280	119.90	47.10	natural	1786.6435	56418.0399	0.1153
Mongolia	812	121.00	50.90	natural	787.7261	23531.0282	0.4367
Mongolia	676	123.20	50.50	natural	356.2728	10095.9196	0.6568
Mongolia	774	121.50	51.30	natural	143.1702	3841.2467	2.0535
Mongolia	810	122.40	50.60	natural	1355.7105	41464.6905	0.2294
Mongolia	887	121.60	50.00	natural	1043.7619	31291.1117	0.3679
Mongolia	660	122.20	52.00	natural	276.0657	7787.6220	1.5576
Mongolia	660	122.20	52.30	natural	193.2160	5209.9614	1.1645
Mongolia	737	120.80	51.30	natural	214.1680	5940.3117	1.5782
Mongolia	846	121.30	49.50	natural	933.1683	27985.1485	0.4040
Shanxi	2212	111.57	37.86	natural	2990.7975	119907.9755	0.0652
Shanxi	2123	111.88	38.67	natural	2736.2482	111001.4104	0.0709
Shanxi	2299	112.04	38.84	natural	1520.9472	63315.1184	0.1098
Shanxi	2278	112.12	38.95	natural	3979.5918	158486.3946	0.0588
Shanxi	2367	113.64	39.16	natural	3013.1004	102366.8122	0.1145
Shanxi	2055	111.46	37.91	natural	2482.8874	108662.1033	0.1607
Shanxi	2300	112.20	39.00	natural	2019.3237	83444.4444	0.2070
Shanxi	1850	111.50	37.83	natural	1962.1422	71772.8532	0.2166
Sichuan	3638	102.40	30.90	natural	10533.3333	388693.3333	0.0375
Sichuan	3600	102.30	30.90	natural	10934.5794	372383.1776	0.0428
Sinkiang	1650	88.10	47.80	natural	203.6247	5536.2473	0.9380
Sinkiang	2100	93.00	43.80	natural	4157.7061	135008.9606	0.1116
Sinkiang	1900	86.40	48.10	natural	4191.9192	108976.1249	0.2178

Sinkiang	1883	86.90	48.00	natural	7733.4630	261994.1634	0.1028
Sinkiang	1700	86.90	47.70	natural	9006.2668	330572.9633	0.1117
Sinkiang	1946	88.70	47.80	natural	3842.2535	109357.7465	0.1775
Sinkiang	1871	89.40	47.00	natural	10673.6243	366574.9526	0.1054
Sinkiang	1989	89.30	47.10	natural	2636.7373	85074.2966	0.3163
Sinkiang	2298	93.10	42.80	natural	8448.1394	242565.3207	0.1263
Yunnan	4240	99.22	28.62	natural	9976.6355	355210.2804	0.0428
**Boreal/alpine Picea-Abies forest**							
Gansu	3280	103.50	34.60	natural	8746.2006	88529.6353	0.2632
Hebei	1536	117.20	42.40	natural	4401.7632	58280.8564	0.1588
Heilongjiang	508	131.80	46.50	natural	4938.0300	82335.2903	0.1533
Heilongjiang	1017	129.40	44.30	natural	19635.4992	307496.0380	0.0631
Heilongjiang	950	124.20	52.60	natural	6720.4696	94057.2267	0.1363
Heilongjiang	410	125.40	52.40	natural	3765.8537	66809.7561	0.1025
Heilongjiang	487	124.50	52.50	natural	3537.5869	62867.9722	0.1583
Heilongjiang	523	124.70	52.30	natural	6702.5641	104948.7179	0.1950
Heilongjiang	800	128.90	47.70	natural	15490.1961	221514.1612	0.0918
Hubei	3300	110.30	31.00	natural	9027.2374	123297.6654	0.1028
Hubei	3260	110.70	32.00	natural	15894.7368	220035.0877	0.0570
Hubei	3200	110.60	31.70	natural	18440.1709	262820.5128	0.0468
Hubei	3200	110.70	31.20	natural	5349.4874	71957.1295	0.1073
Jilin	1286	128.10	42.40	natural	17638.2488	296025.3456	0.0868
Jilin	900	129.70	43.30	natural	15502.7174	235652.1739	0.0736
Mongolia	1300	117.22	43.51	natural	10235.2941	127124.1830	0.0765
Mongolia	2384	105.90	38.77	natural	9685.6810	57211.8743	0.1718
Shanxi	2415	111.83	38.73	natural	5646.0263	75148.7079	0.2051
Shanxi	2475	111.88	38.75	natural	5337.1593	71061.6930	0.1394
Shanxi	2327	111.88	38.77	natural	6985.5072	93739.1304	0.1035
Shanxi	2276	111.93	38.79	natural	4319.8681	57065.1278	0.1213
Shanxi	2243	112.03	38.89	natural	4188.8620	55746.5698	0.1239
Shanxi	2490	113.57	39.12	natural	5525.3490	73490.0808	0.1361
Shanxi	2310	112.20	39.50	natural	3159.4906	41485.7489	0.3298
Shanxi	2680	107.90	33.50	natural	7603.3553	107789.0953	0.1669
Shanxi	2250	108.30	33.30	natural	13628.0488	213460.3659	0.0656
Shanxi	2772	107.70	33.90	natural	9557.6037	134976.9585	0.1085
Shanxi	2770	107.50	33.20	natural	8138.7182	115557.5066	0.1139
Sichuan	3550	98.80	31.20	natural	16461.5385	375288.4615	0.0520
Sichuan	3750	101.60	31.70	natural	20450.2370	738175.3555	0.0422
Sichuan	3675	101.10	31.00	natural	20367.5048	626460.3482	0.0517
Sichuan	3075	103.00	28.80	natural	20706.6381	1383062.0985	0.0467
Sichuan	3480	102.90	32.00	natural	20075.9013	665313.0930	0.0527
Sichuan	3633	101.50	29.00	natural	21838.4401	847771.5877	0.0359
Sichuan	2804	103.10	31.40	natural	19861.3518	556863.0849	0.0577
Sichuan	3000	102.80	31.60	natural	16830.6011	457117.4863	0.0732

Sichuan	3475	102.60	32.00	natural	22685.8513	956834.5324	0.0417
Sichuan	3638	102.20	31.90	natural	21311.9534	820699.7085	0.0343
Sichuan	3500	102.20	31.80	natural	16783.6812	428937.3814	0.1054
Sichuan	3178	103.80	31.60	natural	17687.8613	414523.1214	0.0692
Sichuan	3722	101.20	27.90	natural	16083.7070	394439.4619	0.0669
Sichuan	3200	103.77	33.25	natural	15754.2768	378195.9565	0.0643
Sichuan	3200	103.76	32.60	natural	16156.2500	362546.8750	0.0640
Sichuan	3200	103.75	33.30	natural	15287.3563	326724.1379	0.0696
Sichuan	3200	103.76	33.52	natural	13547.6718	387982.2616	0.0451
Sichuan	3200	104.90	33.52	natural	16490.5660	490150.9434	0.0530
Sichuan	3195	103.67	33.57	natural	16882.5911	472408.9069	0.0494
Sichuan	3200	103.73	33.58	natural	14748.9540	335711.2971	0.0478
Sichuan	3480	103.33	33.60	natural	15691.6427	344178.6744	0.0694
Sichuan	3200	103.74	33.60	natural	12978.7234	311042.5532	0.0470
Sichuan	3252	103.73	33.62	natural	12909.7606	337974.2173	0.0543
Sichuan	3200	103.76	33.63	natural	23538.4615	971730.7692	0.0260
Sichuan	3142	103.75	33.65	natural	15944.4444	460597.2222	0.0720
Sichuan	3204	103.77	33.67	natural	13321.6169	346186.2917	0.0569
Sichuan	3200	104.55	33.55	natural	18709.6774	513241.9355	0.0620
Sichuan	3190	104.66	33.57	natural	14713.3758	387500.0000	0.0628
Sichuan	3200	104.04	33.67	natural	17735.2941	440367.6471	0.0680
Sichuan	3200	104.03	33.01	natural	17094.9721	550782.1229	0.0358
Sichuan	3200	104.10	33.38	natural	12362.0934	227072.1358	0.0707
Sichuan	3200	103.88	33.42	natural	15308.8235	329455.8824	0.0680
Sichuan	3421	103.53	33.47	natural	15366.2182	337967.1151	0.0669
Sichuan	3238	103.70	33.23	natural	16862.7451	458322.4401	0.0459
Sichuan	3200	104.12	33.07	natural	16901.9608	525274.5098	0.0510
Sichuan	3200	104.41	33.60	natural	15020.3252	410853.6585	0.0492
Sichuan	3200	104.05	33.63	natural	15444.4444	417740.7407	0.0540
Sichuan	3200	104.05	33.65	natural	17538.9948	528526.8631	0.0577
Sichuan	3200	104.03	33.68	natural	16341.0853	412868.2171	0.0645
Sichuan	3188	103.92	33.17	natural	15703.4221	410684.4106	0.0526
Sichuan	3200	104.15	33.33	natural	12950.8197	301762.2951	0.0488
Sichuan	3500	103.32	33.38	natural	16444.4444	350185.1852	0.0540
Sichuan	3500	103.34	33.53	natural	15575.6579	347878.2895	0.0608
Sichuan	3500	103.30	33.55	natural	15874.6356	354373.1778	0.0686
Sichuan	3320	103.61	33.32	natural	18051.0441	512227.3782	0.0431
Sichuan	3371	103.47	33.33	natural	16996.9970	389909.9099	0.0666
Sichuan	3500	103.41	33.35	natural	19144.9275	521855.0725	0.0690
Sichuan	3500	103.36	33.37	natural	17793.8517	454068.7161	0.0553
Sichuan	3436	103.50	33.38	natural	15972.9272	363807.1066	0.0591
Sichuan	3500	103.37	33.40	natural	16345.2566	368227.0607	0.0643
Sichuan	3450	103.49	33.43	natural	16888.1119	408496.5035	0.0572
Sichuan	3433	103.46	33.47	natural	17249.5756	452767.4024	0.0589

Sichuan	3433	103.54	33.48	natural	14329.5019	270485.3129	0.0783
Sichuan	3500	103.39	33.62	natural	14192.7711	316337.3494	0.0830
Sichuan	3275	103.66	33.53	natural	18094.3396	499773.5849	0.0530
Sichuan	3500	103.30	33.85	natural	13460.8379	304772.3133	0.0549
Sichuan	3500	103.34	33.76	natural	15085.8034	294617.7847	0.0641
Sichuan	3500	102.93	34.20	natural	12721.3115	240950.8197	0.0915
Sichuan	3500	102.93	34.20	natural	12721.3115	240950.8197	0.0915
Sichuan	3243	103.82	33.27	natural	15729.3869	384799.1543	0.0473
Sichuan	3238	103.66	33.28	natural	13453.4161	235937.8882	0.0805
Sichuan	3500	103.30	33.52	natural	15271.0843	309171.6867	0.0664
Sichuan	3238	103.62	33.55	natural	18185.5670	535010.3093	0.0485
Sichuan	3500	103.32	33.57	natural	16686.9301	406671.7325	0.0658
Sichuan	3500	102.82	34.17	natural	11799.5690	189170.2586	0.0928
Sichuan	3500	103.28	33.58	natural	14756.7568	314445.9459	0.0740
Sichuan	3410	103.57	33.50	natural	15309.8592	311746.4789	0.0710
Sichuan	3500	105.40	33.52	natural	14133.6898	221133.6898	0.1870
Sichuan	3500	103.54	33.52	natural	18078.1250	463875.0000	0.0640
Sichuan	3500	102.92	34.25	natural	12085.7988	199748.5207	0.0676
Sichuan	3450	103.50	33.45	natural	13473.5202	292523.3645	0.0642
Sichuan	3325	103.60	32.60	natural	21452.7845	734309.9274	0.0413
Sichuan	3273	103.55	31.50	natural	16792.4528	158502.3585	0.0848
Sichuan	2900	102.70	30.10	natural	9229.0988	85678.6102	0.1842
Sichuan	3207	103.73	33.07	natural	10637.7079	113354.8983	0.1082
Sichuan	3208	102.33	30.90	natural	19177.0574	680299.2519	0.0401
Sichuan	3800	100.30	30.90	natural	24691.3580	1125135.8025	0.0405
Sichuan	3758	101.00	30.00	natural	17584.8303	433373.2535	0.0501
Sichuan	2805	103.10	30.90	natural	16863.3540	440559.0062	0.0644
Tibet	2750	95.88	29.92	natural	225116.2791	9107790.6977	0.0172
Tibet	3500	97.40	28.60	natural	30168.7764	2071856.5401	0.0237
Tibet	3900	97.10	31.10	natural	12565.7071	168585.7322	0.0799
Tibet	4150	94.70	30.90	natural	23319.6721	605450.8197	0.0244
Tibet	2620	94.90	30.20	natural	16741.2141	316677.3163	0.0313
Tibet	3237	95.70	29.80	natural	21422.9249	1829486.1660	0.0253
Tibet	3900	98.20	31.50	natural	24720.0000	850000.0000	0.0250
Tibet	3750	94.30	31.40	natural	20702.5761	376299.7658	0.0427
Tibet	3800	96.50	31.20	natural	21305.7325	421305.7325	0.0314
Tibet	3982	95.80	30.70	natural	19540.8163	375892.8571	0.0392
Tibet	4180	98.50	29.60	natural	22204.1763	482134.5708	0.0431
Tibet	3999	97.80	29.60	natural	19667.4058	483148.5588	0.0451
Tibet	3642	93.30	29.90	natural	19192.5466	736925.4658	0.0322
Tibet	3631	94.30	29.50	natural	21588.7850	1112429.9065	0.0214
Tibet	3274	94.10	29.20	natural	18805.9701	1033880.5970	0.0268
Tibet	3428	94.10	29.70	natural	23675.2137	1425683.7607	0.0234
Tibet	3540	94.70	29.80	natural	17200.0000	585873.6842	0.0475

Tibet	2590	85.90	28.10	natural	24345.2381	1912738.0952	0.0168
Tibet	2786	85.20	28.90	natural	6238.1853	470283.5539	0.0529
Tibet	3398	85.20	28.90	natural	11155.3785	320996.0159	0.0502
Tibet	3350	85.90	27.90	natural	26480.0000	2026800.0000	0.0125
Tibet	2710	85.90	27.90	natural	19136.3636	1423818.1818	0.0220
Tibet	3835	93.10	29.00	natural	23890.9091	803636.3636	0.0275
Tibet	2000	96.70	28.70	natural	21952.6627	1936094.6746	0.0169
Tibet	3616	97.00	28.40	natural	25203.4884	1046104.6512	0.0344
Tibet	3600	93.80	29.90	natural	17517.7305	922375.8865	0.0282
Sinkiang	2173	88.00	44.20	natural	19286.6407	257380.0259	0.0771
Sinkiang	1870	82.20	43.40	natural	45529.5950	721697.8193	0.0642
Sinkiang	1895	84.60	43.20	natural	81142.1320	1418807.1066	0.0394
Sinkiang	2197	89.10	44.00	natural	35287.5696	513562.1521	0.0539
Sinkiang	2050	86.20	44.30	natural	24402.0101	336864.3216	0.0995
Sinkiang	2210	90.30	43.80	natural	12497.5799	158170.3775	0.1033
Sinkiang	2240	85.60	44.30	natural	2772.8762	27078.3968	0.3967
Sinkiang	2133	85.60	44.30	natural	4123.3407	55752.2124	0.3616
Sinkiang	2500	81.80	43.20	natural	85815.3846	1467415.3846	0.0325
Sinkiang	2285	87.70	43.90	natural	25869.5652	368030.6905	0.0782
Sinkiang	2200	87.13	43.03	natural	13267.8133	178624.0786	0.1221
Sinkiang	2170	88.30	43.30	natural	14665.5377	186104.9958	0.1181
Sinkiang	2283	81.10	43.10	natural	29900.6211	439652.1739	0.0805
Yunnan	3390	98.96	27.45	natural	22025.5183	457783.0941	0.0627
Yunnan	2810	98.96	27.53	natural	18101.0453	277996.5157	0.0574
Yunnan	3590	99.20	27.53	natural	19678.6758	330866.6018	0.1027
Yunnan	3780	99.14	27.82	natural	18848.1338	300913.7709	0.1554
Yunnan	3220	99.26	27.82	natural	26164.8746	1784050.1792	0.0279
Yunnan	3790	99.15	28.00	natural	24482.7586	953029.5567	0.0406
Yunnan	3720	98.96	28.18	natural	26328.1250	2070546.8750	0.0256
Yunnan	3420	98.84	28.53	natural	25405.4054	1110765.7658	0.0222
Yunnan	4120	98.65	28.62	natural	25911.3300	1497142.8571	0.0203
Yunnan	4200	99.02	28.90	natural	20294.6593	356408.8398	0.0543
Yunnan	3720	99.50	26.95	natural	23663.5945	631129.0323	0.0434
Yunnan	3220	99.62	27.02	natural	26225.1656	1879536.4238	0.0151
Yunnan	3240	99.44	27.10	natural	24662.3094	836296.2963	0.0459
Yunnan	3120	99.57	27.17	natural	24778.4200	869460.5010	0.0519
Yunnan	3880	100.20	26.80	natural	21207.2435	434587.5252	0.0497
Yunnan	3820	100.66	27.32	natural	21837.6068	444017.0940	0.0468
Yunnan	3500	100.60	27.53	natural	26200.0000	1829100.0000	0.0200
Yunnan	4100	100.60	27.67	natural	9818.1818	133750.0000	0.0880
Yunnan	3260	99.34	27.36	natural	24904.2146	1072375.4789	0.0261
Yunnan	3559	99.20	27.10	natural	16743.4716	416052.2273	0.0651
Yunnan	3055	99.38	26.14	natural	24939.7590	966947.7912	0.0249
Yunnan	3200	99.70	27.70	natural	22953.2164	839561.4035	0.0342

**Boreal Pinus sylvestris var. mongolica forest**							
Heilongjiang	500	124.70	52.30	natural	9443.1555	295661.2529	0.0431
Heilongjiang	608	126.60	51.70	natural	5122.4707	105665.6017	0.0939
Heilongjiang	560	122.00	52.70	natural	8272.2513	213961.6056	0.0573
Heilongjiang	600	122.40	53.00	natural	8234.3750	219890.6250	0.0640
Heilongjiang	900	124.20	52.60	natural	3752.0000	73488.0000	0.1250
Heilongjiang	890	126.40	50.20	natural	10707.7626	332968.0365	0.0438
Mongolia	832	121.50	50.80	natural	7230.0469	180375.5869	0.0639
Mongolia	793	120.60	52.60	natural	6727.4939	148503.6496	0.0822
Mongolia	633	120.80	52.20	natural	11213.5922	371504.8544	0.0412
Mongolia	700	121.70	50.40	natural	7322.8346	149685.0394	0.0635
**Temperate Pinus tabulaeformis forest**							
An'hui	400	118.00	32.60	natural	2369.5150	29898.3834	0.2165
Sichuan	3200	103.84	33.22	natural	5416.6667	169226.1905	0.0840
Shanxi	1670	108.50	33.33	natural	11329.9492	175553.2995	0.0985
Sichuan	3114	103.79	33.64	natural	5151.8987	166683.5443	0.0790
Shanxi	1520	107.30	34.00	natural	16910.2990	926079.7342	0.0301
Shanxi	1589	112.12	35.43	natural	2960.3960	32287.1287	0.1010
Shanxi	1242	112.02	35.58	natural	5736.8421	65828.9474	0.0760
Shanxi	1150	112.08	35.60	natural	5703.8835	65497.5728	0.0824
Shanxi	1180	112.10	35.68	natural	2572.6141	27775.9336	0.1205
Shanxi	990	112.22	35.72	natural	2295.7461	24550.9791	0.1481
Shanxi	1120	112.17	35.75	natural	4359.3750	49000.0000	0.0640
Shanxi	980	112.28	35.77	natural	1910.5691	20111.7886	0.0984
Shanxi	1350	113.11	35.95	natural	10554.2169	127698.7952	0.0830
Shanxi	1300	112.06	35.98	natural	4203.6554	47056.1358	0.1532
Shanxi	1200	112.40	36.00	natural	6099.2908	70449.1726	0.0423
Shanxi	1380	111.07	36.08	natural	7240.2044	84752.9813	0.0587
Shanxi	1100	112.29	36.17	natural	3288.3642	36163.5750	0.1186
Shanxi	1250	112.47	36.17	natural	5815.6912	66824.4085	0.0803
Shanxi	1150	112.16	36.32	natural	2821.2040	30601.9766	0.1113
Shandong	1000	117.20	36.40	natural	2122.8070	26216.3743	0.1710
Shanxi	1065	112.25	36.42	natural	6333.7547	73312.2630	0.0791
Shanxi	1332	112.47	36.42	natural	4742.6982	53630.0417	0.0719
Shanxi	1900	111.87	36.43	natural	8424.9471	100000.0000	0.0946
Shanxi	1550	111.22	36.45	natural	2279.2608	24380.5613	0.1461
Shanxi	1440	111.36	36.45	natural	4317.5997	23644.3469	0.2733
Shanxi	1135	112.29	36.45	natural	8325.4344	98704.5814	0.0633
Shanxi	1130	112.41	36.47	natural	5802.9979	66723.7687	0.0934
Shanxi	1210	112.43	36.48	natural	5029.9401	57245.5090	0.0668
Shanxi	1250	112.45	36.48	natural	3119.0476	34178.5714	0.0840
Shandong	680	118.20	36.50	natural	2841.2256	28941.5042	0.2513
Shanxi	1153	109.40	36.50	natural	6114.7327	153220.3390	0.0767
Shanxi	1197	112.18	36.52	natural	4498.7775	50599.0220	0.0818

Shanxi	1300	112.54	36.52	natural	4663.6086	52737.0031	0.0654
Shanxi	1260	112.30	36.53	natural	4981.3433	56604.4776	0.1072
Shanxi	1345	112.43	36.53	natural	9750.7788	117320.8723	0.0321
Shanxi	1290	112.41	36.55	natural	3397.9475	37434.4356	0.0877
Shanxi	1650	111.20	36.58	natural	5045.2781	57328.5899	0.1546
Shanxi	1450	112.10	36.58	natural	7378.6408	86650.4854	0.0412
Shanxi	1550	112.07	36.60	natural	11253.4060	136948.2289	0.0367
Shanxi	1207	112.34	36.60	natural	2805.5965	30441.8262	0.1358
Shanxi	1580	111.22	36.63	natural	3372.0930	37148.1028	0.1634
Shanxi	1680	112.07	36.63	natural	12334.6304	151400.7782	0.0514
Shanxi	1251	112.34	36.63	natural	2725.2747	29571.4286	0.0910
Shanxi	1800	112.10	36.65	natural	7750.5330	91268.6567	0.0938
Shanxi	1210	112.27	36.65	natural	5094.8509	57949.4128	0.1107
Shanxi	1790	112.07	36.70	natural	9741.0604	117065.3514	0.0811
Shanxi	1804	112.12	36.70	natural	16322.4181	205440.8060	0.0397
Shandong	250	121.10	36.70	natural	1027.4554	11616.1902	0.3533
Shanxi	1445	112.00	36.70	natural	1018.1049	10581.9422	0.8506
Shanxi	1352	112.23	36.73	natural	5533.4282	63328.5917	0.0703
Shanxi	1352	112.34	36.77	natural	7926.4556	93626.1491	0.0979
Shanxi	1322	112.37	36.77	natural	2685.8214	29088.0700	0.1601
Shanxi	1810	112.12	36.78	natural	7523.9923	88416.5067	0.1042
Shanxi	1600	112.17	36.82	natural	8676.8150	103278.6885	0.0854
Shanxi	1527	112.34	36.82	natural	6023.3298	69501.5907	0.0943
Shanxi	1421	112.41	36.83	natural	9796.6728	117763.4011	0.0541
Shanxi	1610	113.04	36.85	natural	9748.2838	117185.3547	0.0874
Shanxi	1583	112.37	36.90	natural	3776.6714	41941.6785	0.1406
Shanxi	1470	112.41	36.90	natural	3625.5768	40131.8392	0.1517
Shanxi	1820	112.04	36.92	natural	3722.0259	41272.0848	0.1698
Shanxi	1600	112.30	36.92	natural	2287.2340	24445.2888	0.1316
Shanxi	1680	112.39	36.92	natural	8373.8796	99321.3828	0.0781
Shanxi	1720	112.35	36.93	natural	3769.6850	41865.1575	0.2032
Shanxi	1819	112.30	36.97	natural	4295.1542	48193.8326	0.1362
Shanxi	1781	112.35	36.97	natural	11866.0287	145263.1579	0.0418
Shanxi	1850	112.28	36.98	natural	5546.4160	63525.2644	0.0851
Shanxi	1813	112.37	36.98	natural	4282.5112	48082.9596	0.0892
Shanxi	1820	112.30	37.00	natural	5329.1039	60888.1840	0.1261
Shanxi	1670	112.35	37.00	natural	6726.3427	78286.4450	0.0782
Shanxi	240	120.00	37.00	natural	434.1085	4287.5264	0.7095
Shanxi	1690	112.33	37.02	natural	3673.9380	40734.7876	0.0871
Shanxi	1865	112.37	37.02	natural	5000.0000	56725.0674	0.0742
Shanxi	1630	112.42	37.02	natural	6452.5140	74818.4358	0.0716
Shanxi	1600	113.45	37.12	natural	7606.9731	89413.6292	0.0631
Shanxi	1856	113.52	37.18	natural	7066.4928	82620.5997	0.0767
Shanxi	275	121.70	37.20	natural	1676.2854	20687.3033	0.3812

Shanxi	1320	112.51	37.23	natural	3127.5510	34209.1837	0.1960
Shanxi	1500	111.23	37.25	natural	5551.0480	28999.3239	0.1479
Shanxi	1700	113.59	37.27	natural	5639.1876	64671.4456	0.0837
Shanxi	1530	111.18	37.28	natural	3906.1851	21457.0361	0.2409
Shanxi	1395	111.23	37.30	natural	3489.1254	19504.8589	0.2161
Shanxi	1551	113.48	37.32	natural	8205.6075	97084.1121	0.0535
Shanxi	1520	113.37	37.33	natural	4207.3171	47103.6585	0.0984
Shanxi	1550	111.32	37.35	natural	2581.2712	27831.1499	0.2061
Shanxi	1445	113.48	37.40	natural	5965.2174	68817.3913	0.0575
Shanxi	1490	113.46	37.42	natural	4160.0000	46595.5556	0.1125
Shanxi	1575	111.77	37.47	natural	4214.0791	47184.1851	0.1037
Shanxi	1650	111.81	37.47	natural	18158.6402	230849.8584	0.0353
Shanxi	1430	113.31	37.47	natural	6504.6296	75462.9630	0.0864
Shanxi	1590	113.35	37.47	natural	7332.1555	85918.7279	0.0566
Shanxi	1595	111.70	37.47	natural	11334.3328	138140.9295	0.0667
Shanxi	1641	113.28	37.47	natural	7674.4186	90342.7173	0.0817
Shanxi	1582	113.33	37.47	natural	7574.6714	89080.0478	0.0837
Shanxi	1600	113.27	37.47	natural	12557.7558	154471.9472	0.0606
Shanxi	1620	111.75	37.48	natural	15987.6543	200843.6214	0.0486
Shanxi	1750	111.59	37.50	natural	19372.1973	247847.5336	0.0223
Shanxi	1680	111.72	37.50	natural	24346.1538	318384.6154	0.0260
Shanxi	1460	111.86	37.50	natural	3869.9690	43095.9752	0.1292
Shanxi	1645	111.66	37.52	natural	12308.6124	153373.2057	0.0836
Shanxi	1820	111.66	37.63	natural	5954.6539	68591.8854	0.0838
Shanxi	1680	111.70	37.63	natural	6064.0920	69983.5661	0.1217
Shanxi	1424	111.75	37.63	natural	9957.4468	119914.8936	0.0940
Shanxi	1300	111.84	37.63	natural	4439.5410	49920.5649	0.1133
Shanxi	1750	111.59	37.65	natural	20162.7486	258915.0090	0.0553
Shanxi	1575	111.63	37.65	natural	13325.8929	164799.1071	0.0448
Shanxi	1300	111.73	37.65	natural	9347.8261	111992.7536	0.0552
Shanxi	1450	111.82	37.65	natural	10726.9155	129901.7682	0.0509
Shanxi	1800	111.57	37.67	natural	23361.7021	304510.6383	0.0235
Shanxi	1230	111.97	37.67	natural	9801.5123	49829.8677	0.1058
Shanxi	1360	111.82	37.68	natural	5132.4965	58410.0418	0.0717
Shanxi	1985	111.43	37.70	natural	30202.8986	403420.2899	0.0345
Shanxi	1550	111.66	37.70	natural	7307.2061	85663.7168	0.0791
Shanxi	1420	111.70	37.70	natural	6209.2624	71801.0292	0.0583
Shanxi	1670	111.50	37.72	natural	20983.6066	270601.0929	0.0549
Shanxi	1600	111.86	37.72	natural	6884.1545	80252.9960	0.0751
Shanxi	1100	113.59	37.72	natural	5360.8247	61340.2062	0.0485
Shanxi	1670	111.48	37.73	natural	28904.1096	384452.0548	0.0146
Shanxi	1600	111.61	37.73	natural	9215.6863	110150.8296	0.0663
Shanxi	1650	111.66	37.73	natural	10698.6900	129636.0990	0.0687
Shanxi	1410	111.70	37.73	natural	14181.3602	176297.2292	0.0397

Shanxi	1700	111.84	37.73	natural	13904.7619	172452.3810	0.0420
Shanxi	1940	111.64	37.75	natural	12289.5126	150856.7208	0.0677
Shanxi	1560	111.79	37.75	natural	6922.0246	80793.4337	0.0731
Shanxi	1400	111.82	37.75	natural	12773.9726	157363.0137	0.0292
Shanxi	1550	111.86	37.75	natural	9792.5311	117821.5768	0.0482
Shanxi	1555	111.91	37.75	natural	8956.2764	106784.2031	0.0709
Shanxi	1750	111.95	37.78	natural	3548.3871	39242.9230	0.1519
Shanxi	1740	111.70	37.80	natural	10049.5050	121039.6040	0.0404
Shanxi	1580	111.98	37.80	natural	4424.4984	49746.5681	0.0947
Shanxi	1475	112.32	37.82	natural	4863.2580	55112.9608	0.0841
Shanxi	1711	111.36	37.90	natural	9048.5830	108076.9231	0.0494
Shanxi	1692	111.50	37.90	natural	4583.4716	53305.6754	0.3013
Shanxi	1497	111.98	37.92	natural	3431.2878	37856.5255	0.1157
Shanxi	1400	112.98	38.02	natural	2475.1656	26572.8477	0.1208
Shanxi	1680	111.25	38.10	natural	8628.8089	102576.1773	0.0722
Shanxi	1390	112.39	38.13	natural	7815.7895	92197.3684	0.0760
Shanxi	1667	111.32	38.22	natural	9244.2882	110527.2408	0.0569
Shanxi	1750	111.37	38.25	natural	18637.8738	237740.8638	0.0301
Shanxi	1940	111.43	38.38	natural	6020.4082	69500.0000	0.0980
Shanxi	1200	112.40	38.42	natural	2828.5256	30697.1154	0.1248
Shanxi	1620	111.37	38.43	natural	7366.2967	86417.7598	0.0991
Shanxi	1650	111.39	38.45	natural	9437.5000	113187.5000	0.0800
Shanxi	1690	111.73	38.62	natural	5982.6947	68974.0420	0.0809
Shanxi	1700	111.97	38.68	natural	2807.9393	30461.1792	0.1713
Shanxi	1700	111.94	38.68	natural	6523.4375	75742.1875	0.1024
Beijing	553	116.80	40.30	natural	7145.7627	38271.1864	0.1475
Hebei	350	118.90	40.40	natural	2512.7802	19284.3099	0.2543
Liaoning	600	123.20	40.50	natural	7367.6782	90063.5145	0.1417
Hebei	1100	117.90	40.90	natural	4970.6458	44155.2511	0.1533
Hebei	898	118.60	41.00	natural	8072.4638	76376.8116	0.1380
Mongolia	1110	119.30	41.60	natural	2307.4636	37600.3568	0.3363
Hebei	1140	117.30	42.00	natural	17281.8312	187281.8312	0.0699
Mongolia	843	118.97	42.27	natural	3234.8020	64595.6497	0.1793
Jilin	560	129.50	42.60	natural	2479.7268	31323.0901	0.2343
**Temperate mixed coniferous-broadleaved forest**							
Heilongjiang	300	127.20	50.70	planted	1068.6778	7984.5956	0.6232
Heilongjiang	400	129.70	44.70	natural	21111.1111	897111.1111	0.0270
Heilongjiang	350	129.00	47.00	planted	3200.0000	95245.4545	0.1100
Heilongjiang	378	131.80	46.50	natural	14657.9805	630651.4658	0.0307
Heilongjiang	264	133.50	46.30	natural	17489.3617	892297.8723	0.0235
Heilongjiang	240	132.90	45.80	natural	17150.0000	1035500.0000	0.0200
Heilongjiang	450	130.18	45.18	planted	1507.7178	11460.8600	0.3628
Heilongjiang	600	130.20	44.50	natural	13775.0000	601900.0000	0.0400
Heilongjiang	400	128.50	44.30	natural	9607.2508	295045.3172	0.0662

Heilongjiang	514	129.40	44.30	natural	16306.3063	558828.8288	0.0444
Heilongjiang	300	127.50	45.20	planted	3105.7764	30082.5206	0.2666
Heilongjiang	403	129.30	48.10	natural	11140.0000	474060.0000	0.0500
Jilin	650	128.20	42.42	natural	6433.7568	247994.5554	0.1102
Jilin	770	128.33	42.50	natural	10434.7826	606750.5721	0.0437
Jilin	250	125.30	43.90	planted	7519.8588	90406.0018	0.1133
Jilin	400	125.42	42.52	planted	2572.3906	26632.9966	0.2970
Jilin	696	128.20	43.30	natural	13950.3386	587878.1038	0.0443
Jilin	600	127.20	42.30	natural	6728.0453	258286.1190	0.0706
Jilin	300	127.00	41.90	planted	4408.4507	46136.1502	0.2130
Jilin	675	128.10	42.40	natural	8806.6826	279653.9379	0.0838
Liaoning	233	123.90	40.88	planted	13076.9231	150809.1908	0.1001
Liaoning	500	125.30	41.60	planted	2143.7126	18158.6826	0.3340
**Temperate typical deciduous broadleaved forest**							
Beijing	1365	115.43	39.97	natural	7386.0705	96173.6887	0.1163
Guizhou	540	108.12	28.25	natural	3377.2652	65271.8287	0.1214
Hebei	1300	117.90	40.90	natural	685.7143	21184.4156	0.3850
Hebei	1200	118.40	40.60	natural	1052.8097	35829.3839	0.2954
Hebei	1150	118.60	41.00	natural	1609.4822	51915.1591	0.1603
Hebei	1100	118.70	40.50	natural	381.9361	12511.4100	0.8326
Hebei	1005	117.40	40.60	natural	1717.6634	81293.4631	0.1438
Heilongjiang	293	130.50	45.70	natural	1021.6718	31037.1517	0.2584
Heilongjiang	350	126.60	51.70	natural	8480.3922	204803.9216	0.0408
Heilongjiang	177	133.50	46.30	natural	5039.3013	146899.5633	0.1145
Heilongjiang	190	132.90	45.80	natural	4128.7284	166656.2009	0.0637
Heilongjiang	218	134.00	46.80	natural	9689.7375	325489.2601	0.0419
Heilongjiang	492	127.50	45.20	natural	4746.5438	111990.7834	0.1085
Heilongjiang	400	127.60	45.43	natural	3566.6667	127475.0000	0.1200
Heilongjiang	500	128.20	45.00	natural	6745.3416	165838.5093	0.0805
Heilongjiang	383	127.60	44.90	natural	3397.3866	108047.6556	0.1301
Heilongjiang	500	131.80	46.50	natural	5008.0000	146960.0000	0.0625
Heilongjiang	240	130.70	47.00	natural	10715.6309	247476.4595	0.0531
Jilin	550	128.10	42.40	natural	7755.6818	560568.1818	0.0352
Jilin	800	127.50	40.70	natural	13456.5217	520000.0000	0.0460
Jilin	650	128.20	43.30	natural	30353.9823	863716.8142	0.0226
Jilin	615	129.00	42.50	natural	2216.2577	67346.6258	0.1304
Jilin	700	126.90	41.80	natural	7068.4039	517035.8306	0.0307
Jilin	650	130.20	44.50	natural	8400.8097	223117.4089	0.0494
Jilin	450	129.70	43.30	natural	10447.2272	311788.9088	0.0559
Jilin	450	129.70	43.30	natural	1325.4237	41010.1695	0.2950
Jilin	450	129.50	42.60	natural	427.2743	13345.2021	0.5617
Jilin	450	126.70	43.50	natural	750.0000	23950.0000	0.3200
Jilin	450	126.70	43.50	natural	489.0720	12581.5120	0.5582
Jilin	460	126.70	42.90	natural	2389.3333	78533.3333	0.1875

Liaoning	350	125.00	41.70	natural	7712.8548	252136.8948	0.0599
Liaoning	400	124.70	40.70	natural	753.1447	13402.5157	0.6360
Liaoning	350	121.70	42.00	natural	1333.3333	56413.0435	0.1380
Mongolia	498	124.00	50.40	natural	4923.9544	133840.3042	0.0526
Ningxia	2050	106.27	35.60	natural	3385.0000	48825.0000	0.2000
Shandong	400	117.90	35.70	natural	6612.1495	131425.2336	0.0428
Shandong	700	117.20	36.40	natural	2701.0309	47505.1546	0.1455
Shanxi	1250	111.41	34.97	natural	9260.2740	130986.3014	0.0730
Shanxi	1100	111.42	34.98	natural	2605.2819	48336.9022	0.1401
Shanxi	936	111.55	35.03	natural	5401.1461	65279.3696	0.1396
Shanxi	830	111.66	35.13	natural	2164.2764	33168.1877	0.1534
Shanxi	1100	111.57	35.15	natural	5842.0441	116573.7515	0.0861
Shanxi	1050	111.57	35.20	natural	4833.3333	65755.5556	0.0900
Shanxi	1490	112.32	35.23	natural	4147.8439	53855.2361	0.1948
Shanxi	1350	112.30	35.25	natural	8384.7981	106389.5487	0.0421
Shanxi	1500	112.45	35.27	natural	10745.5013	135681.2339	0.0389
Shanxi	800	111.79	35.30	natural	2057.5080	31015.9744	0.1565
Shanxi	1422	112.19	35.30	natural	6511.2406	125753.5387	0.1201
Shanxi	850	111.99	35.32	natural	2815.6863	51725.4902	0.1275
Shanxi	1180	111.44	35.33	natural	6824.3243	88952.7027	0.1184
Shanxi	1200	111.99	35.35	natural	1439.2523	24747.6636	0.2140
Shanxi	1220	111.79	35.37	natural	6934.8010	88518.2049	0.1181
Shanxi	1380	111.68	35.38	natural	3947.9167	74713.5417	0.1920
Shanxi	1675	112.01	35.40	natural	7826.8551	155918.7279	0.0566
Shanxi	1500	111.79	35.45	natural	7075.9717	88533.5689	0.1132
Shanxi	1214	112.08	35.47	natural	3276.6338	62345.5685	0.1117
Shanxi	1300	111.84	35.48	natural	7245.6576	147220.8437	0.0403
Shanxi	1280	113.16	35.52	natural	1215.2466	20448.4305	0.2230
Shanxi	1280	113.29	35.52	natural	9115.6463	113551.0204	0.0735
Shanxi	1500	111.84	35.55	natural	1431.8574	24197.8148	0.1739
Shanxi	1250	112.15	35.63	natural	9871.1340	122525.7732	0.0776
Shanxi	1300	112.15	35.67	natural	4034.9345	54681.2227	0.1145
Shanxi	1385	113.52	35.67	natural	6329.9663	89006.7340	0.0594
Shanxi	1400	112.04	35.72	natural	3479.6574	47071.7345	0.1868
Shanxi	1500	110.98	35.82	natural	7862.9032	99677.4194	0.0496
Shanxi	1247	110.00	35.97	natural	2556.7239	44786.9397	0.1807
Shanxi	1440	110.96	36.10	natural	7372.2045	88107.0288	0.1252
Shanxi	1300	111.07	36.12	natural	6982.9060	87786.3248	0.1170
Shanxi	1630	110.91	36.13	natural	3410.8527	46887.2987	0.1677
Shanxi	1490	111.02	36.15	natural	11349.8099	141730.0380	0.0526
Shanxi	1620	111.04	36.17	natural	7453.3107	97249.5756	0.0589
Shanxi	1510	110.64	36.27	natural	4100.9682	57655.6017	0.1446
Shanxi	1410	110.96	36.27	natural	13376.2887	162345.3608	0.0388
Shanxi	1270	111.67	36.30	natural	5094.9367	65759.4937	0.0632

Shanxi	1400	111.29	36.33	natural	3681.0631	47222.5914	0.1505
Shanxi	1450	111.87	36.37	natural	3619.4416	49369.1830	0.1934
Shanxi	1400	110.60	36.38	natural	3796.3945	75026.5111	0.0943
Shanxi	1900	111.29	36.42	natural	15553.3981	189320.3883	0.0515
Shanxi	1281	111.94	36.42	natural	10272.7273	126181.8182	0.0660
Shanxi	1015	111.94	36.45	natural	24389.5349	295290.6977	0.0344
Shanxi	1450	111.91	36.47	natural	7557.7558	97227.7228	0.0909
Shanxi	1633	111.85	36.48	natural	8896.7136	172370.8920	0.0426
Shanxi	1350	111.94	36.48	natural	5088.2353	67156.8627	0.1020
Shanxi	1500	111.92	36.52	natural	2602.2305	37486.9888	0.1345
Shanxi	1380	112.00	36.52	natural	1979.3564	29987.8567	0.1647
Shanxi	1400	111.92	36.55	natural	4800.2160	61177.1058	0.1852
Shanxi	1490	111.96	36.55	natural	3150.6849	43822.5701	0.1533
Shanxi	1460	111.94	36.57	natural	6291.6667	81486.1111	0.0720
Shanxi	1580	111.22	36.60	natural	3348.5454	63456.9310	0.1753
Shanxi	1450	111.94	36.60	natural	2627.3585	48995.2830	0.2120
Shanxi	1630	112.01	36.62	natural	7420.5268	96158.0381	0.1101
Shanxi	1670	113.33	36.63	natural	1955.1438	33866.4066	0.2051
Shanxi	1930	111.92	36.65	natural	14116.6937	177277.1475	0.0617
Shanxi	1650	111.27	36.67	natural	4033.7838	53013.5135	0.1480
Shanxi	1650	111.94	36.67	natural	6634.6922	84700.2398	0.1251
Shanxi	1770	112.07	36.67	natural	4299.9336	58652.9529	0.1507
Shanxi	1760	112.07	36.67	natural	10486.7257	130383.4808	0.0678
Shanxi	1700	112.10	36.68	natural	6172.5293	81088.7772	0.1194
Shanxi	1630	113.30	36.68	natural	6520.1123	85724.9766	0.1069
Shanxi	1740	113.28	36.70	natural	4924.6231	96231.1558	0.0796
Shanxi	1630	111.18	36.70	natural	14650.7666	183117.5468	0.0587
Shanxi	1290	111.29	36.73	natural	3198.6242	42381.7713	0.1163
Shanxi	1761	112.10	36.73	natural	14023.2558	173255.8140	0.0430
Shanxi	1699	111.99	36.75	natural	8049.0196	102117.6471	0.1020
Shanxi	1579	112.48	36.75	natural	12247.9339	151421.4876	0.0605
Shanxi	1650	111.20	36.77	natural	8620.2186	110450.8197	0.0732
Shanxi	2011	111.96	36.77	natural	3909.0445	52682.6776	0.1957
Shanxi	1811	112.05	36.77	natural	7012.1951	88609.7561	0.0820
Shanxi	1608	112.14	36.77	natural	4544.9735	60185.1852	0.1890
Shanxi	1605	112.17	36.78	natural	5852.3985	76553.5055	0.1355
Shanxi	1550	111.20	36.80	natural	23396.8254	291396.8254	0.0315
Shanxi	1703	111.92	36.80	natural	9362.2142	118555.9567	0.0831
Shanxi	1640	113.33	36.82	natural	8707.8652	112865.1685	0.0534
Shanxi	1470	111.20	36.83	natural	7807.6923	101833.3333	0.0780
Shanxi	1540	113.35	36.85	natural	6068.5484	123447.5806	0.0496
Shanxi	1200	110.71	36.87	natural	1483.1349	26408.7302	0.2016
Shanxi	1640	111.11	36.90	natural	5415.9445	71819.7574	0.1154
Shanxi	1750	111.99	36.90	natural	3881.5789	52286.1842	0.1216

Shanxi	2150	112.08	36.97	natural	7968.0000	102544.0000	0.0625
Shanxi	1555	111.11	37.03	natural	10620.6897	129448.2759	0.0870
Shanxi	1690	111.11	37.05	natural	5847.0336	77090.7791	0.1399
Shanxi	1150	111.29	37.05	natural	5369.4581	66472.9064	0.1015
Shanxi	1556	113.50	37.05	natural	13243.2432	165210.2102	0.0666
Shanxi	1430	111.31	37.07	natural	7314.8148	95532.4074	0.0864
Shanxi	1820	111.11	37.12	natural	7717.2875	98892.0726	0.1047
Shanxi	1750	111.20	37.12	natural	3535.9526	48287.6205	0.1349
Shanxi	1340	111.38	37.12	natural	5379.6095	68459.8698	0.0922
Shanxi	1482	111.38	37.20	natural	12125.8503	148605.4422	0.0588
Shanxi	1520	111.38	37.30	natural	3097.5610	41699.1870	0.1230
Shanxi	1780	111.38	37.37	natural	6806.3872	87704.5908	0.1002
Shanxi	1840	111.34	37.40	natural	17590.3614	217108.4337	0.0166
Shanxi	1500	113.35	37.43	natural	3711.3402	49436.9548	0.1261
Shanxi	1690	111.72	37.47	natural	3191.9092	44849.5313	0.2027
Shanxi	1620	111.75	37.47	natural	3333.3333	46886.5122	0.1683
Shanxi	1712	111.43	37.48	natural	3128.4530	44730.6630	0.1448
Shanxi	1620	111.79	37.48	natural	3458.1712	46687.7432	0.2056
Shanxi	1490	111.84	37.48	natural	5492.6387	72763.3069	0.0883
Shanxi	1550	111.90	37.48	natural	2581.7676	46666.6667	0.1437
Shanxi	1575	113.35	37.50	natural	4047.4244	53589.5339	0.1223
Shanxi	1484	111.79	37.52	natural	3724.2128	71824.1042	0.0921
Shanxi	1420	111.88	37.52	natural	3537.0370	47268.5185	0.1080
Shanxi	1670	111.68	37.57	natural	7328.9597	92380.5061	0.1067
Shanxi	1452	111.70	37.58	natural	8548.1240	106623.1648	0.0613
Shanxi	1650	111.88	37.63	natural	4649.3131	59826.4642	0.1383
Shanxi	1810	111.45	37.67	natural	7823.0088	103150.4425	0.0565
Shanxi	1500	111.88	37.67	natural	8967.0330	111626.3736	0.0455
Shanxi	1022	111.48	37.70	natural	11380.2817	143873.2394	0.0710
Shanxi	1691	111.84	37.70	natural	5234.3059	67621.5738	0.1131
Shanxi	1630	111.66	37.77	natural	4862.8258	63237.3114	0.1458
Shanxi	2020	111.75	37.77	natural	7724.6496	96438.5820	0.1213
Shanxi	1900	111.84	37.77	natural	4774.7748	63186.9369	0.1776
Shanxi	1880	111.91	37.78	natural	3599.4707	48989.8544	0.2267
Shanxi	1621	111.32	37.90	natural	3767.6349	50141.0788	0.1205
Shanxi	2120	111.48	38.07	natural	7296.5116	96744.1860	0.0688
Shanxi	1630	112.30	38.10	natural	4923.4694	61658.1633	0.0784
Shanxi	1410	112.41	38.12	natural	2869.5234	39817.0438	0.2077
Shanxi	1710	111.32	38.18	natural	11163.9344	145737.7049	0.0610
Shanxi	1990	111.34	38.20	natural	2584.0038	37737.8135	0.2113
Shanxi	2117	112.06	38.80	natural	2911.3067	37772.5118	0.1477
Shanxi	1785	111.10	37.50	natural	10179.1045	188611.9403	0.0670
Shanxi	1810	112.30	36.50	natural	6519.6078	119566.9935	0.1224
Shanxi	1400	112.00	36.70	natural	3397.8495	54822.5806	0.1860

Shanxi	1400	112.00	36.70	natural	6737.2881	138771.1864	0.0472
Sichuan	2160	104.20	33.20	natural	8093.3333	157266.6667	0.0750
Sichuan	2600	103.00	32.60	natural	9918.5889	198561.7368	0.0737
**Temperate/subtropical montane Populus-Betula deciduous forest**							
Beijing	1500	115.60	39.80	natural	863.2479	20555.5556	0.2340
Beijing	1492	115.43	39.97	natural	4539.5349	78576.7442	0.1075
Guizhou	680	107.85	27.78	natural	3913.7380	105031.9489	0.0626
Guizhou	1800	105.90	26.70	natural	5871.6981	141426.4151	0.1325
Guizhou	748	108.09	25.75	natural	9530.3867	304654.6961	0.0724
Hebei	1170	117.90	40.90	natural	2203.8981	54722.6387	0.2001
Hebei	1600	115.20	40.90	natural	3729.4686	108753.6232	0.1035
Hebei	1300	116.30	41.20	natural	1514.7059	39936.2745	0.2040
Hebei	1400	118.40	40.60	natural	4807.0175	144128.6550	0.0855
Hebei	1260	117.20	41.60	natural	2038.4615	55493.5897	0.1560
Hebei	1600	117.60	41.90	natural	3049.1803	86743.1694	0.0915
Hebei	1600	117.30	42.00	natural	1443.0894	37808.9431	0.2460
Heilongjiang	350	130.50	45.70	natural	9633.2046	310772.2008	0.0518
Heilongjiang	450	129.70	44.70	natural	7685.0095	253624.2884	0.0527
Heilongjiang	400	128.40	44.30	natural	11141.4392	360967.7419	0.0403
Heilongjiang	261	126.60	51.70	natural	1579.9373	41724.1379	0.1595
Heilongjiang	507	126.60	51.70	natural	3976.5458	119275.0533	0.0938
Heilongjiang	400	124.70	52.00	natural	7270.9552	179210.5263	0.1026
Heilongjiang	226	133.50	46.30	natural	12474.0484	260882.3529	0.0578
Heilongjiang	880	130.20	46.30	natural	7429.6675	226445.0128	0.0782
Heilongjiang	800	130.20	44.50	natural	14728.2609	484402.1739	0.0184
Heilongjiang	650	129.20	43.80	natural	6087.5000	160775.0000	0.0800
Heilongjiang	170	134.00	46.80	natural	34763.7795	612874.0157	0.0254
Heilongjiang	450	128.20	45.00	natural	4823.6092	95820.8955	0.1474
Heilongjiang	455	127.55	45.32	natural	1710.6549	82815.2493	0.2046
Heilongjiang	500	124.50	52.50	natural	9040.0000	147260.0000	0.0500
Heilongjiang	533	127.60	44.90	natural	8437.5000	197731.4815	0.0864
Heilongjiang	700	128.40	49.50	natural	6944.0459	211032.9986	0.0697
Heilongjiang	600	129.00	47.00	natural	17954.5455	393333.3333	0.0396
Heilongjiang	400	131.80	46.50	natural	12063.8821	333882.0639	0.0407
Jilin	600	128.27	42.75	natural	1988.0716	29973.4924	0.3018
Jilin	590	128.10	42.40	natural	3614.8848	86284.7017	0.1693
Jilin	845	126.90	41.80	natural	12997.2376	284558.0110	0.0724
Jilin	817	128.10	43.50	natural	993.5484	19022.2222	0.6975
Jilin	650	128.80	42.40	natural	2759.4937	60898.7342	0.3160
Jilin	550	126.40	41.90	natural	4106.7961	82718.4466	0.1030
Jilin	600	125.70	42.20	natural	5413.7931	112413.7931	0.1450
Jilin	550	126.90	44.40	natural	13138.1381	292897.8979	0.0666
Jilin	590	127.20	44.00	natural	2388.4157	51649.0630	0.2935
Jilin	650	129.70	43.30	natural	5972.6688	147652.7331	0.1244

Jilin	800	129.20	43.30	natural	7394.0021	182368.1489	0.0967
Jilin	700	129.40	42.70	natural	12692.3077	238447.8022	0.0728
Jilin	847	129.50	42.60	natural	2633.4647	63321.3902	0.2791
Jilin	650	126.70	43.50	natural	1448.5981	31310.8706	0.4066
Jilin	684	127.10	42.90	natural	7241.0546	174943.5028	0.1062
Liaoning	500	119.40	42.00	natural	4959.2476	105962.3824	0.1595
Mongolia	712	121.50	50.80	natural	4112.5828	91403.9735	0.1510
Mongolia	1750	119.00	42.30	natural	273.2240	14153.0055	0.7320
Mongolia	1434	119.30	41.60	natural	2152.4202	52615.8599	0.2913
Mongolia	150	122.00	49.50	natural	9395.8076	236806.4118	0.0811
Mongolia	677	124.00	50.40	natural	3080.8081	91893.9394	0.1188
Mongolia	800	126.60	51.70	natural	9081.8859	300049.6278	0.0403
Mongolia	800	109.40	40.70	natural	1454.7591	30495.8872	0.4255
Mongolia	600	122.10	48.50	natural	3968.1668	115060.3732	0.1822
Ningxia	2125	106.27	35.60	natural	1385.0000	35035.0000	0.2000
Qinghai	2650	101.80	37.00	natural	3388.6085	23594.0880	0.2774
Shanxi	1838	111.98	35.43	natural	2589.5876	48519.2698	0.1479
Shanxi	1675	111.99	35.47	natural	4034.2679	79018.6916	0.0642
Shanxi	1590	111.02	36.18	natural	782.4989	19225.9150	0.2377
Shanxi	1700	111.00	36.27	natural	2020.4082	54989.7959	0.0980
Shanxi	1420	111.89	36.53	natural	7826.0870	248586.9565	0.0460
Shanxi	1980	112.05	36.65	natural	2661.4699	74665.9243	0.0898
Shanxi	1480	113.30	36.65	natural	3329.6582	61835.7222	0.1814
Shanxi	1900	112.05	36.80	natural	1525.2153	40184.5018	0.1626
Shanxi	2210	112.03	36.86	natural	4892.9664	148226.2997	0.0654
Shanxi	1550	113.36	36.87	natural	2461.9517	81996.4190	0.1117
Shanxi	1750	112.08	36.97	natural	3183.7607	98824.7863	0.0936
Shanxi	1910	111.23	37.08	natural	1725.6011	46315.4173	0.1414
Shanxi	1845	113.50	37.12	natural	2008.0591	66783.0759	0.1489
Shanxi	2000	113.55	37.20	natural	2385.5165	66240.6816	0.0939
Shanxi	1680	111.14	37.22	natural	5434.1737	164789.9160	0.0357
Shanxi	1659	111.41	37.42	natural	2887.5380	96068.8956	0.0987
Shanxi	1880	111.57	37.48	natural	2936.5854	99453.6585	0.1025
Shanxi	1810	111.68	37.48	natural	5856.7775	211163.6829	0.0782
Shanxi	1772	111.57	37.52	natural	1234.8574	46889.4099	0.2559
Shanxi	1750	111.66	37.55	natural	4259.8870	121841.8079	0.0885
Shanxi	1925	111.63	37.57	natural	2439.8625	67617.4112	0.0873
Shanxi	1550	111.39	37.67	natural	2063.1720	80026.8817	0.1488
Shanxi	2230	111.52	37.67	natural	3648.3740	105975.6098	0.0984
Shanxi	1805	111.86	37.68	natural	3194.6755	91198.0033	0.0601
Shanxi	1447	111.52	37.70	natural	3425.4992	98955.4531	0.0651
Shanxi	2100	111.45	37.72	natural	6298.5075	194835.8209	0.0335
Shanxi	1952	111.45	37.75	natural	5649.8674	172068.9655	0.0377
Shanxi	1965	111.57	37.75	natural	4782.6087	146585.3659	0.0943

Shanxi	1798	111.48	37.77	natural	1939.2917	71039.9101	0.1779
Shanxi	2050	111.59	37.78	natural	5484.8967	167011.1288	0.0629
Shanxi	1810	111.39	37.85	natural	2632.5247	88095.2381	0.1113
Shanxi	1850	111.36	37.93	natural	4643.7346	157248.1572	0.0814
Shanxi	2000	111.57	37.93	natural	4496.5278	133975.6944	0.0576
Shanxi	1947	111.59	37.93	natural	2648.8352	74219.1544	0.1159
Shanxi	1799	111.55	37.97	natural	1823.1047	48916.9675	0.1108
Shanxi	1680	111.43	37.98	natural	1449.8807	42440.3341	0.1676
Shanxi	1780	111.32	38.08	natural	5114.2132	152043.1472	0.0788
Shanxi	1751	111.32	38.25	natural	5384.6154	163333.3333	0.0585
Shanxi	2078	111.43	38.32	natural	4612.5461	137546.1255	0.0542
Shanxi	1990	111.39	38.35	natural	4215.2466	124633.7818	0.0669
Shanxi	1888	111.43	38.35	natural	3315.2502	95376.7330	0.1659
Shanxi	1580	111.37	38.47	natural	2205.8824	71078.4314	0.1224
Shanxi	1970	111.62	38.52	natural	2313.9881	63861.6071	0.1344
Shanxi	2020	111.78	38.65	natural	4089.7098	127770.4485	0.0758
Shanxi	2030	112.95	39.30	natural	2820.5128	79446.6937	0.0741
Shanxi	2010	113.97	39.93	natural	943.2421	23666.8028	0.2449
Shanxi	2637	107.90	33.50	natural	1255.0996	32843.7725	0.4167
Shanxi	2060	106.10	33.30	natural	12597.0149	243940.2985	0.0335
Shanxi	2137	108.30	33.30	natural	2773.7226	69169.0062	0.1781
Shanxi	1950	108.40	33.40	natural	10555.5556	177870.3704	0.0432
Shanxi	2210	108.50	33.50	natural	4113.6364	107522.7273	0.1320
Shanxi	1700	108.00	33.50	natural	13656.2500	463812.5000	0.0320
Shanxi	1700	107.50	33.20	natural	3308.6420	94921.8107	0.1215
Sichuan	3200	104.19	32.98	natural	8960.0000	252920.0000	0.0500
Sichuan	3300	98.80	31.20	natural	3152.1739	72995.6522	0.2300
Sichuan	2580	102.70	30.60	natural	5411.9318	166235.7955	0.0704
Sichuan	2600	103.10	31.40	natural	7958.3746	237770.0694	0.1009
Sichuan	3200	104.83	33.50	natural	3230.7692	89884.6154	0.0780
Sichuan	3200	104.91	33.53	natural	5679.4872	156858.9744	0.0780
Sichuan	3180	103.69	33.57	natural	6408.3333	138158.3333	0.1200
Sichuan	3200	103.77	33.33	natural	8835.8209	196014.9254	0.0670
Sichuan	2850	102.30	30.90	natural	7511.0457	236642.1208	0.0679
Sichuan	2185	103.10	30.90	natural	9162.7907	273000.0000	0.0430
Tibet	2300	95.00	30.10	natural	21391.1290	581370.9677	0.0496
Tibet	3355	93.30	29.50	natural	4151.7857	125803.5714	0.0672
Tibet	3500	85.20	28.90	natural	12416.1074	416107.3826	0.0149
Tibet	2335	96.70	28.70	natural	24100.7194	605323.7410	0.0278
Tibet	2010	97.00	28.40	natural	3064.3612	63924.1114	0.2082
Yunnan	3012	99.20	27.10	natural	5486.2658	161343.7268	0.1347
Yunnan	3640	107.80	27.10	natural	4240.0000	140275.5556	0.2250
Yunnan	3400	99.70	27.70	natural	1014.8125	25694.9259	0.3173
**Desert riverside woodland**							

Sinkiang	900	80.80	38.40	natural	2000.0000	126242.8571	0.0700
Sinkiang	900	88.10	40.10	natural	4200.0000	329400.0000	0.0100
Sinkiang	900	80.60	40.40	natural	5250.0000	418083.3333	0.0120
Sinkiang	925	85.20	41.50	natural	2487.5000	74037.5000	0.0800
Sinkiang	900	82.90	41.70	natural	7000.0000	555142.8571	0.0140
Sinkiang	900	80.80	40.40	natural	1004.0161	66224.8996	0.0498
Sinkiang	950	84.30	41.70	natural	1469.9332	119665.9243	0.0449
Sinkiang	650	86.20	44.30	natural	951.7872	34904.4057	0.2406
Sinkiang	500	86.40	48.00	natural	5632.1839	178007.6628	0.0261
**Subtropical mixed evergreen-deciduous broadleaved forest**							
Fujian	1330	117.30	27.10	natural	5160.9756	122736.5854	0.2050
Fujian	470	117.30	27.30	natural	8417.8322	130052.4476	0.1144
Guizhou	1093	106.70	26.40	natural	5977.6536	169056.4867	0.1611
Guizhou	1580	107.00	28.20	natural	7114.5686	174389.4389	0.2121
Guizhou	600	107.98	25.25	natural	1886.6667	104066.6667	0.1500
Guizhou	1985	106.80	28.10	natural	7710.4785	220423.9855	0.1651
Guizhou	2150	104.20	26.80	natural	2046.8998	37110.4928	0.2516
Guizhou	1420	108.00	26.00	natural	2086.9565	46060.8696	0.2300
Henan	1296	111.60	33.60	natural	3549.4505	74258.2418	0.1820
Henan	1080	111.30	33.60	natural	2128.4501	39973.4607	0.1884
Henan	500	114.80	31.60	natural	2344.6328	46065.3753	0.2478
Henan	1142	111.70	33.50	natural	4212.5000	85562.5000	0.1600
Hubei	1827	110.60	31.70	natural	7142.8571	159730.6122	0.1225
Hubei	1600	110.70	32.00	natural	6396.8411	131510.3653	0.1013
Hubei	1730	110.30	31.13	natural	4499.3234	108322.0568	0.1478
Hunan	500	110.40	29.10	natural	4972.2222	84944.4444	0.1080
Hunan	1210	110.10	29.30	natural	15231.9109	362059.3692	0.0539
Shanxi	1800	108.20	33.00	natural	18504.3478	290086.9565	0.0575
Sichuan	1635	106.80	32.30	natural	6838.4279	168829.6943	0.1145
Tibet	2075	96.70	28.70	natural	30465.9498	1333763.4409	0.0279
Yunnan	2518	99.20	27.10	natural	4439.9460	168697.7058	0.1482
Yunnan	2600	99.70	27.70	natural	6687.9489	237797.2865	0.1253
**Subtropical evergreen broadleaved forest**							
Fujian	400	116.30	25.80	natural	6158.4158	122000.0000	0.1515
Fujian	400	119.30	26.00	natural	36153.8462	883020.6379	0.0533
Fujian	400	117.20	24.70	natural	11968.3544	248329.1139	0.1580
Fujian	870	118.10	27.00	natural	4317.3913	87673.9130	0.2300
Fujian	380	118.10	27.10	natural	16510.6383	333540.4255	0.1175
Fujian	650	118.70	26.30	natural	5235.0877	104487.7193	0.2850
Fujian	860	118.00	26.70	natural	151333.3333	3768400.0000	0.0150
Fujian	1100	118.10	26.60	natural	1466.6667	37127.7778	0.5400
Fujian	550	118.50	27.60	natural	5373.7043	112089.4708	0.1833
Fujian	423	117.40	26.10	natural	36240.0000	880288.0000	0.0625
Fujian	750	116.40	25.00	natural	9055.1839	183720.7358	0.1196

Fujian	750	117.10	27.20	natural	11762.2610	261438.0715	0.1203
Fujian	793	117.60	27.10	natural	7394.3925	146844.8598	0.2675
Fujian	438	117.40	27.30	natural	54700.2398	1209088.7290	0.0417
Fujian	415	117.60	27.00	natural	50207.8522	1144711.3164	0.0433
Fujian	450	117.50	25.20	natural	10460.0000	209746.6667	0.1500
Guangdong	270	112.58	23.13	natural	18556.5476	633139.8810	0.0672
Guangdong	80	110.00	20.70	natural	16085.7143	196523.8095	0.1050
Guangdong	380	111.88	23.45	natural	3979.6737	94549.3447	0.3739
Guangdong	400	115.60	22.90	natural	17276.1905	361923.8095	0.0525
Guangdong	500	114.00	24.90	natural	5264.2416	104337.6802	0.1457
Guangxi	500	110.82	24.83	natural	2740.9091	100981.8182	0.2200
Guangxi	1002	108.62	25.10	natural	17183.6735	371292.5170	0.0735
Guangxi	820	109.97	25.45	natural	13675.0000	288550.0000	0.0800
Guangxi	1085	109.93	25.45	natural	6906.5232	139361.1298	0.1487
Guangxi	1680	109.98	25.60	natural	16838.7097	355580.6452	0.0620
Guangxi	550	110.64	25.17	natural	13907.8156	284989.9800	0.0499
Guangxi	835	110.28	25.67	natural	11592.4276	240835.1893	0.0898
Guangxi	450	110.28	25.97	natural	1992.5611	45961.7428	0.1882
Guangxi	500	110.10	25.90	natural	3792.7873	78493.2763	0.3272
Guangxi	700	108.36	23.57	natural	6117.5236	67208.8143	0.1906
Guangxi	700	109.63	25.10	natural	26583.1435	573986.3326	0.0439
Guangxi	1160	107.71	21.85	natural	4806.9936	50978.6558	0.4404
Guangxi	1550	106.34	24.45	natural	27993.1973	767823.1293	0.0294
Guangxi	1978	106.40	24.52	natural	13630.9524	361646.8254	0.0504
Guangxi	1100	104.41	24.23	natural	4783.4101	111244.2396	0.1085
Guangxi	1172	104.68	24.58	natural	5112.4260	128000.0000	0.0845
Guangxi	305	109.93	25.17	natural	2284.3701	51014.1140	0.1913
Guangxi	468	109.75	25.38	natural	18931.4195	393110.0478	0.0627
Guangxi	500	110.60	26.00	natural	16615.3846	370717.9487	0.0585
Guangxi	560	110.00	25.70	natural	16329.3468	357010.1196	0.1087
Guangxi	700	110.60	26.00	natural	1678.5714	28208.3333	0.1680
Guizhou	965	106.00	24.75	natural	4982.3633	122345.6790	0.1134
Guizhou	540	105.80	25.00	natural	24367.6471	763941.1765	0.0680
Guizhou	1543	105.87	28.32	natural	2597.4982	57954.3782	0.2718
Guizhou	1400	105.70	28.32	natural	3198.9597	75123.5371	0.1538
Guizhou	1073	105.83	28.35	natural	2326.5742	52801.4941	0.1874
Guizhou	800	106.20	28.53	natural	1754.6778	37367.9834	0.2405
Guizhou	840	106.16	28.65	natural	2520.7861	59051.3983	0.2646
Guizhou	750	108.45	25.50	natural	2253.1646	51417.7215	0.2765
Guizhou	1200	108.33	25.58	natural	1401.9746	31743.3004	0.3545
Guizhou	1400	108.29	25.62	natural	5638.4181	143175.1412	0.0885
Guizhou	560	107.97	26.20	natural	3338.3234	71347.3054	0.2004
Guizhou	892	108.00	26.23	natural	1078.7081	21640.0125	0.6378
Guizhou	1080	107.93	26.27	natural	1912.5249	41602.3857	0.2515

Guizhou	900	107.97	26.30	natural	2737.3237	59370.9493	0.2623
Guizhou	1415	107.44	29.22	natural	1751.1067	39837.6783	0.2033
Guizhou	1280	107.71	28.68	natural	2205.9349	45770.4684	0.2797
Guizhou	1340	107.68	25.90	natural	6242.2635	152298.8506	0.1131
Guizhou	1900	104.57	27.13	natural	7447.6987	184748.9540	0.0478
Guizhou	1190	107.63	26.95	natural	2829.9204	68324.8009	0.3516
Guizhou	870	108.55	26.45	natural	3895.7346	92559.2417	0.1055
Guizhou	700	108.83	26.52	natural	2362.2181	55738.5111	0.5897
Guizhou	700	108.71	26.73	natural	24278.9598	663144.2080	0.0423
Guizhou	860	109.39	26.55	natural	1619.7416	36767.1414	0.9057
Guizhou	950	107.98	26.52	natural	2505.2720	56242.0919	0.2371
Guizhou	1600	108.16	26.33	natural	3561.9940	85262.0366	0.2347
Guizhou	1430	108.27	26.42	natural	3063.1527	70004.9727	0.2011
Guizhou	1865	108.23	26.52	natural	3820.5980	90730.8970	0.1505
Guizhou	915	108.20	26.40	natural	7307.6923	177224.0803	0.1196
Guizhou	1785	108.00	26.30	natural	8809.5238	227823.1293	0.0882
Guizhou	490	108.92	25.95	natural	3072.8825	73000.6566	0.1523
Guizhou	780	108.96	25.95	natural	2659.5745	60494.3680	0.1598
Guizhou	800	109.36	26.02	natural	6798.6799	164174.9175	0.1212
Guizhou	780	109.40	26.08	natural	2194.3973	50382.0034	0.2356
Guizhou	670	109.28	26.20	natural	3866.5526	87647.5620	0.1169
Guizhou	750	109.32	26.23	natural	2077.9934	47638.2278	0.5462
Guizhou	800	107.79	25.21	natural	4012.5000	94156.2500	0.1600
Guizhou	800	107.91	25.21	natural	3448.1062	78165.2730	0.2033
Guizhou	815	107.95	25.21	natural	1980.4699	42648.7641	0.3277
Guizhou	750	107.99	25.21	natural	3101.7573	70498.5697	0.2447
Guizhou	790	107.95	25.25	natural	2089.4006	46065.0186	0.2953
Guizhou	850	107.99	25.25	natural	2901.0239	64618.8851	0.2637
Guizhou	900	107.87	25.30	natural	3038.9817	67549.7216	0.1257
Guizhou	953	107.91	25.33	natural	2574.7508	57296.5116	0.2408
Guizhou	600	108.06	25.37	natural	1260.1969	26334.7398	0.3555
Guizhou	650	108.14	25.37	natural	1717.1717	36310.7263	0.2079
Guizhou	700	107.99	25.40	natural	3333.3333	82559.1398	0.1395
Guizhou	760	108.02	25.40	natural	1310.2119	27055.8767	0.2595
Guizhou	970	107.67	25.43	natural	2363.7138	56379.8978	0.2348
Guizhou	850	108.14	25.43	natural	2496.3645	57716.9171	0.2063
Guizhou	700	107.79	25.47	natural	2323.8950	52796.9613	0.2896
Guizhou	800	108.10	25.52	natural	1882.0786	40176.5490	0.3002
Guizhou	800	108.14	25.52	natural	2150.7863	46951.8964	0.2162
Guizhou	1500	107.04	25.55	natural	1282.0513	25817.0685	0.2613
Guizhou	1000	107.88	25.55	natural	1640.0426	33874.3344	0.4695
Guizhou	650	107.92	25.55	natural	3529.4118	81880.4243	0.1037
Guizhou	540	107.96	25.55	natural	4484.1675	101103.1665	0.1958
Guizhou	850	108.05	25.58	natural	1853.9326	38968.3350	0.1958

Guizhou	700	108.09	25.58	natural	4275.3623	96280.1932	0.1242
Guizhou	1462	106.97	26.37	natural	12573.1895	328382.1263	0.0649
Guizhou	710	106.96	25.43	natural	6049.5283	148360.8491	0.0848
Guizhou	1090	107.13	26.02	natural	3309.3525	74532.3741	0.1668
Guizhou	754	108.05	25.75	natural	2281.4070	52517.5879	0.1990
Guizhou	1032	108.12	26.02	natural	3310.6961	77674.0238	0.1767
Guizhou	620	108.20	26.02	natural	8328.2365	206085.6269	0.0981
Guizhou	810	107.93	26.02	natural	4781.7558	108813.1437	0.2039
Guizhou	950	108.05	27.17	natural	2078.3373	43377.2982	0.2502
Guizhou	980	108.45	27.53	natural	9320.6951	235418.6414	0.0633
Guizhou	850	109.25	28.13	natural	5679.5132	140507.0994	0.0986
Guizhou	1619	108.31	26.55	natural	3134.0405	77531.3404	0.2074
Guizhou	500	109.35	26.73	natural	4034.2429	90117.7100	0.1869
Guizhou	700	109.02	27.10	natural	19727.8912	532891.1565	0.0588
Guizhou	420	109.34	27.70	natural	7086.3971	168630.5147	0.1088
Guizhou	885	106.12	25.03	natural	6618.8769	166045.4002	0.0837
Guizhou	680	106.24	25.07	natural	18561.6438	478664.3836	0.0292
Guizhou	740	107.23	27.02	natural	1834.2800	41132.6428	0.2993
Guizhou	1030	107.63	27.02	natural	2685.3984	62441.2713	0.2171
Guizhou	1210	107.59	27.05	natural	1976.1800	43242.1703	0.2267
Guizhou	1030	107.67	27.13	natural	2231.3204	51069.6008	0.1954
Guizhou	1500	106.70	27.10	natural	12738.6692	328341.3693	0.1037
Guizhou	1120	106.03	28.35	natural	3355.0186	81821.5613	0.1076
Guizhou	1180	106.03	28.40	natural	3735.4651	80283.4302	0.1376
Guizhou	1320	106.07	28.40	natural	3072.7273	73000.0000	0.1650
Guizhou	1100	106.07	28.43	natural	3212.5604	79130.4348	0.1242
Guizhou	1380	106.19	28.43	natural	1743.5321	35753.6558	0.3556
Guizhou	1360	106.32	28.50	natural	4548.6111	109222.2222	0.1440
Guizhou	990	106.24	28.53	natural	5680.6084	140501.9011	0.1315
Guizhou	1120	106.28	28.53	natural	3072.5943	72999.4373	0.1777
Guizhou	1210	106.40	28.57	natural	3072.5943	72999.4373	0.1777
Guizhou	1460	106.48	28.57	natural	1761.6034	42410.3376	0.1896
Guizhou	1186	104.61	25.75	natural	7102.9836	175909.5284	0.1039
Guizhou	740	108.43	28.25	natural	4442.1199	106338.9121	0.1434
Guizhou	727	108.14	28.68	natural	2228.7145	47992.4875	0.2396
Guizhou	760	108.19	28.97	natural	1311.3855	27111.1111	0.3645
Guizhou	1030	108.60	27.97	natural	1666.0733	35382.6985	0.2809
Guizhou	1010	108.30	27.17	natural	2065.4149	52337.9770	0.1651
Guizhou	1160	106.36	25.40	natural	4916.3347	118342.6295	0.1255
Guizhou	1072	108.65	27.90	natural	4001.6779	98292.7852	0.2384
Guizhou	851	108.13	25.77	natural	3754.6012	84539.8773	0.1630
Guizhou	864	108.53	25.90	natural	3076.3689	72997.1182	0.1388
Guizhou	645	108.32	25.98	natural	4041.1523	94032.9218	0.1215
Guizhou	1290	108.60	26.08	natural	5364.5485	123525.0836	0.1495

Guizhou	600	108.48	26.30	natural	4091.3508	100029.1545	0.1029
Guizhou	710	108.47	26.42	natural	2450.7874	54335.6299	0.2032
Hunan	1390	110.16	26.18	natural	2241.3793	50240.6609	0.2784
Hunan	650	110.20	26.35	natural	4792.8026	97502.7263	0.1834
Hunan	870	110.56	26.53	natural	5078.0829	104119.5477	0.1857
Hunan	1150	110.20	26.20	natural	26318.9270	618196.7213	0.0671
Hunan	450	109.75	26.83	natural	13537.7778	309653.3333	0.1125
Hunan	1120	111.55	24.70	natural	59409.4488	1505472.4409	0.0254
Hunan	550	111.70	24.90	natural	29171.7791	672085.8896	0.0978
Hunan	910	110.96	25.02	natural	12410.4859	249520.4604	0.1564
Hunan	1332	111.95	25.20	natural	19271.1864	406135.5932	0.0590
Hunan	650	111.36	25.50	natural	5793.5200	117935.2004	0.1821
Hunan	650	111.30	25.20	natural	6208.4257	128203.9911	0.1353
Hunan	500	109.51	26.62	natural	8533.9943	172507.0822	0.1412
Hunan	700	112.80	24.90	natural	10677.7778	224777.7778	0.0900
Hunan	510	113.78	25.50	natural	4390.9586	89217.2457	0.2389
Hunan	1440	110.13	29.78	natural	2857.5320	61329.3381	0.3671
Hunan	430	111.76	25.88	natural	2836.6317	60845.9449	0.2577
Hunan	870	110.12	26.43	natural	8938.4289	180859.8726	0.0942
Hunan	740	110.12	26.72	natural	11178.3439	229466.5605	0.1256
Hunan	768	110.36	26.72	natural	4118.0203	84302.0305	0.1576
Hunan	860	110.12	26.78	natural	7621.8487	153436.9748	0.1190
Hunan	880	110.19	26.93	natural	8197.4922	163385.5799	0.1914
Hunan	390	110.90	29.10	natural	5107.4956	116647.2981	0.1721
Hunan	580	109.84	26.35	natural	2201.0399	49569.6129	0.3462
Hunan	1260	110.60	26.70	natural	12361.6734	276504.7233	0.0741
Hunan	1050	112.86	24.95	natural	2503.5162	54989.4515	0.2844
Hunan	1110	112.74	24.98	natural	3511.2564	72730.5737	0.2754
Hunan	910	112.82	24.98	natural	1796.0763	42594.6394	0.3619
Hunan	1480	112.98	24.98	natural	3565.1007	74204.0268	0.3725
Hunan	600	110.26	28.73	natural	3320.2568	69418.6876	0.2804
Hunan	570	110.26	28.82	natural	4123.3244	83667.5603	0.1865
Hunan	570	113.36	26.25	natural	9054.7703	188454.0636	0.1132
Hunan	700	110.47	28.13	natural	13501.5448	282409.8867	0.0971
Hunan	740	110.59	28.23	natural	4842.3773	97317.8295	0.1935
Hunan	670	110.63	28.27	natural	5027.7778	100597.2222	0.1440
Hunan	410	113.69	28.38	natural	2463.8193	54182.8450	0.2833
Hunan	633	113.68	26.25	natural	5574.7410	111687.2225	0.2027
Hunan	550	113.64	26.28	natural	5002.1863	101836.4670	0.2287
Hunan	1300	113.76	26.10	natural	5470.5882	109470.5882	0.1530
Hunan	1200	113.84	26.25	natural	6408.8670	127704.4335	0.2030
Hunan	680	113.64	26.35	natural	6880.4665	143542.2741	0.1372
Hunan	575	113.73	26.43	natural	11277.0563	234033.1890	0.1386
Hunan	250	114.05	26.50	natural	3395.4727	71181.7577	0.3004

Hunan	827	113.70	26.50	natural	11597.0962	252749.5463	0.1102
Hunan	500	112.30	28.80	natural	1838.8489	43738.1295	0.3475
Hunan	360	114.02	28.23	natural	1405.7101	36117.4759	0.5499
Hunan	1040	114.15	28.48	natural	2969.2058	63076.1750	0.3085
Jiangxi	600	114.40	26.50	natural	9630.9524	188654.7619	0.1680
Jiangxi	688	114.50	28.40	natural	1589.1865	25156.6448	0.3958
Jiangxi	601	114.50	28.40	natural	11131.1054	251593.8303	0.0778
Jiangxi	483	114.60	28.40	natural	19865.0927	429005.0590	0.0593
Jiangxi	538	114.70	28.30	natural	16057.8035	352693.6416	0.0865
Jiangxi	813	114.70	28.30	natural	1164.8250	25209.4843	0.4344
Sichuan	1900	103.40	28.30	natural	40372.3404	1181968.0851	0.0188
Sichuan	1520	103.50	28.80	natural	17618.1474	464404.5369	0.0529
Sichuan	1200	106.39	28.64	natural	9286.5780	216517.5333	0.0827
Tibet	1900	97.40	28.60	natural	8625.0000	236401.7857	0.1120
Tibet	2670	94.90	30.20	natural	15916.9550	607785.4671	0.0289
Tibet	2980	85.20	28.90	natural	3577.8175	116994.6333	0.1118
Tibet	2020	97.00	28.40	natural	8104.8387	306431.4516	0.0992
Tibet	2020	85.90	27.90	natural	6234.1463	247892.6829	0.1025
Yunnan	2823	99.32	27.53	natural	7663.3663	304811.8812	0.1010
Yunnan	3385	98.96	27.97	natural	6768.0180	241689.1892	0.0888
Yunnan	2550	101.00	24.53	natural	1820.0000	245585.0000	0.2000
Yunnan	2394	99.68	25.28	natural	3467.9021	119497.0218	0.1511
Yunnan	2488	99.73	25.28	natural	8122.2222	328644.4444	0.0900
Yunnan	2645	99.68	25.43	natural	8370.4235	308033.0223	0.1393
Yunnan	3460	98.78	28.10	natural	3224.0964	117195.1807	0.2075
Yunnan	3340	98.90	28.18	natural	6537.7176	239825.9188	0.1034
Yunnan	4160	99.02	28.75	natural	2810.0775	92577.5194	0.2064
Yunnan	3135	99.56	26.08	natural	5222.9299	189851.3800	0.0942
Yunnan	2480	99.62	26.08	natural	5004.4366	149103.8154	0.1127
Yunnan	3450	100.10	26.37	natural	34297.1888	1069076.3052	0.0249
Yunnan	2783	100.34	26.52	natural	4056.6038	138799.3139	0.1166
Yunnan	2040	101.19	26.45	natural	4779.3190	149489.2812	0.1586
Yunnan	2903	99.56	26.23	natural	17869.1983	542911.3924	0.0474
Yunnan	3060	99.80	26.67	natural	3922.6974	141167.7632	0.1216
Yunnan	2860	100.35	27.02	natural	7874.8524	261109.7993	0.0847
Yunnan	2860	99.44	27.17	natural	25309.0333	793280.5071	0.0631
Yunnan	2780	99.75	27.17	natural	11875.0000	415777.0270	0.0592
Yunnan	3240	100.23	27.17	natural	12969.3487	480000.0000	0.0522
Yunnan	2900	99.44	27.38	natural	3506.3663	126513.2223	0.1021
Yunnan	3300	100.89	26.73	natural	6908.8812	241914.6482	0.0867
Yunnan	2920	100.72	27.17	natural	7539.2670	224149.2147	0.0764
Yunnan	2570	100.90	27.32	natural	5115.6069	177061.6570	0.1038
Yunnan	3280	100.54	27.67	natural	2633.0832	82100.7503	0.2799
Yunnan	2900	98.78	25.58	natural	18415.1786	652857.1429	0.0448

Yunnan	930	100.85	23.20	natural	3083.7438	78300.4926	0.2030
Yunnan	2850	99.92	25.58	natural	6859.8383	270040.4313	0.0742
Yunnan	2526	99.14	25.50	natural	6526.1514	234441.8423	0.1281
Yunnan	2790	99.08	25.80	natural	6612.9032	221388.8889	0.1116
Yunnan	2240	99.38	26.23	natural	7040.6732	246353.4362	0.0713
Zhejiang	275	120.17	30.25	natural	2906.9069	64498.4985	0.1665
Zhejiang	438	119.27	29.48	natural	1511.7801	29018.3246	0.4584
**Sclerophyllous evergreen Quercus forest**							
Sichuan	3800	101.50	29.00	natural	8402.3669	259664.6943	0.1014
Sichuan	3800	101.20	27.90	natural	9059.2334	279895.4704	0.1148
Tibet	3000	97.40	28.60	natural	19002.6247	670419.9475	0.0381
Tibet	3281	96.10	29.70	natural	20100.7557	624836.2720	0.0397
Tibet	3020	95.70	29.80	natural	16723.3010	555024.2718	0.0412
Tibet	2925	94.30	29.50	natural	13101.4493	456405.7971	0.0345
Tibet	2375	85.90	27.90	natural	29333.3333	967636.3636	0.0165
Tibet	3103	91.90	27.90	natural	38793.9698	1464924.6231	0.0199
Yunnan	3400	99.70	27.70	natural	10581.0056	266446.9274	0.0895
**Tropical rainforest and monsoon forest**							
Hainan	790	109.82	19.23	natural	7763.9752	508296.3620	0.1127
Hainan	15	110.73	19.50	planted	3965.4296	45526.1820	0.1967
Hainan	15	110.62	19.88	planted	10572.7273	375172.7273	0.1100
Guangxi	10	109.40	21.40	natural	11234.2857	132754.2857	0.0875
Guangxi	15	109.72	21.47	planted	950.0000	39148.6486	0.7400
Yunnan	585	110.42	21.68	natural	4372.0930	78217.0543	0.1290
Guangxi	20	109.60	21.70	natural	74800.0000	1772433.3333	0.0300
Guangxi	10	108.30	21.70	natural	13066.6667	164939.3939	0.1650
Fujian	15	117.92	24.40	planted	294.7115	7691.3462	2.0800
Hainan	875	108.68	18.62	natural	7809.6677	256621.3494	0.1986
Hainan	679	108.80	18.70	natural	6305.8590	322323.7339	0.1007
Guangxi	35	109.10	21.50	planted	6823.3618	76880.3419	0.1404
Guangxi	28	108.70	21.85	natural	7257.6636	82228.6661	0.1207
**Subtropical montane Pinus yunnanensis and P. khasya forest**							
Tibet	2200	97.40	28.60	natural	36491.2281	2116257.3099	0.0171
Yunnan	2636	99.08	26.15	natural	57303.9216	1028676.4706	0.0204
Yunnan	2880	99.32	26.30	natural	108988.7640	2063595.5056	0.0089
Yunnan	3050	99.32	27.82	natural	7550.1114	120133.6303	0.0898
Yunnan	2020	99.38	25.72	natural	29756.9444	508854.1667	0.0288
Yunnan	2715	99.38	26.02	natural	18625.0000	309550.0000	0.0400
Yunnan	2940	99.44	27.23	natural	8640.1674	138190.3766	0.0956
Yunnan	2940	99.44	27.32	natural	47238.8060	834776.1194	0.0134
Yunnan	2550	99.50	25.72	natural	106732.6733	2017128.7129	0.0101
Yunnan	2980	99.50	27.17	natural	23753.2134	400282.7763	0.0389
Yunnan	3040	99.57	27.32	natural	142747.2527	2768131.8681	0.0091
Yunnan	2104	99.62	25.43	natural	3569.9039	55885.8058	0.1874

Yunnan	2340	99.62	25.58	natural	9537.8151	153277.3109	0.0714
Yunnan	2635	99.62	25.87	natural	56517.4129	1020845.7711	0.0201
Yunnan	3120	99.68	26.80	natural	1766.7436	27780.6005	0.4330
Yunnan	2540	99.69	27.10	natural	12960.7843	211156.8627	0.0510
Yunnan	2900	99.70	27.70	natural	1138.3260	18225.5507	0.5675
Yunnan	2092	99.74	25.37	natural	46530.6122	821700.6803	0.0147
Yunnan	2750	99.74	25.43	natural	8249.5667	131698.4402	0.1154
Yunnan	2400	99.74	25.87	natural	15567.6856	255873.3624	0.0458
Yunnan	2780	99.75	26.95	natural	8492.9577	135760.5634	0.0710
Yunnan	1950	99.79	25.28	natural	27927.2727	475818.1818	0.0275
Yunnan	1800	99.80	25.58	natural	12545.8248	203930.7536	0.0491
Yunnan	1951	99.85	25.28	natural	22018.9274	369305.9937	0.0317
Yunnan	2800	100.20	26.80	natural	7357.0948	125299.2603	0.1487
Yunnan	2210	100.34	26.37	natural	11849.1921	158671.4542	0.0557
Yunnan	3060	100.48	27.45	natural	2048.1530	32110.8179	0.3032
Yunnan	3300	100.54	27.75	natural	16528.5996	272781.0651	0.0507
Yunnan	2540	100.59	27.10	natural	78581.3149	1447266.4360	0.0289
Yunnan	2940	100.60	27.82	natural	8941.3265	143214.2857	0.0784
Yunnan	2730	100.65	27.10	natural	5522.0522	87056.7057	0.2222
Yunnan	3278	100.66	27.67	natural	14733.1787	241438.5151	0.0431
Yunnan	2779	100.70	26.70	natural	21200.0000	374433.3333	0.0600
Yunnan	3110	100.72	27.32	natural	4375.0000	68659.7938	0.1552
Yunnan	2735	100.76	26.45	natural	16497.5845	272101.4493	0.0414
Yunnan	2850	100.89	26.45	natural	105605.0955	1994140.1274	0.0157
Yunnan	2241	101.20	26.60	natural	12413.4078	218547.4860	0.0895
Sichuan	2850	101.20	27.90	natural	8743.9614	150712.5604	0.0828
Yunnan	2039	101.34	26.73	natural	21074.2188	357871.0938	0.0512
Yunnan	2210	101.70	26.00	natural	12472.6477	213205.6893	0.0914
Sichuan	2068	102.50	28.00	natural	3566.6667	52672.2222	0.1800
Guizhou	2344	104.13	26.77	natural	828.8438	13564.0282	0.7239
Guizhou	1697	104.41	26.73	natural	12747.8261	171165.2174	0.0575
Guangxi	970	106.23	24.73	natural	70896.5517	1294000.0000	0.0145
Guangxi	1011	106.29	24.80	natural	62212.3894	1123716.8142	0.0113
Guangxi	206	106.29	24.95	natural	53553.7190	955785.1240	0.0121
**Subtropical Pinus massoniana forest**							
Guangxi	175	110.65	23.00	planted	18066.5281	258336.7983	0.0481
Guangdong	200	113.70	23.00	planted	7750.2296	119274.5638	0.1089
Guangdong	270	112.35	23.08	planted	7016.0000	86452.0000	0.2500
Guangdong	500	115.10	23.60	planted	7633.8028	117596.2441	0.1065
Guangxi	300	105.08	23.75	planted	20689.6552	465747.1264	0.0435
Guangxi	380	111.77	24.23	natural	13856.0886	203210.3321	0.0542
Guangxi	255	111.77	24.45	planted	17637.3626	252912.0879	0.0546
Guangxi	215	108.28	24.58	planted	14388.8889	210388.8889	0.0540
Jiangxi	600	114.50	24.70	planted	11656.1404	164343.8596	0.1425

Guangxi	490	108.80	24.80	planted	13770.4918	202071.5350	0.0671
Jiangxi	600	114.40	24.80	planted	11726.3158	165263.1579	0.1425
Hunan	380	111.30	25.30	natural	18134.9911	660071.0480	0.0563
Guizhou	1390	105.13	25.58	planted	5719.2076	95951.7657	0.1161
Fujian	820	116.90	25.70	natural	5755.2743	191409.2827	0.1185
Guangxi	500	110.60	25.80	natural	9716.1290	147032.2581	0.0775
Guangxi	500	110.10	25.90	natural	15468.1648	455205.9925	0.0534
Guizhou	1050	106.65	26.23	natural	9723.2704	237270.4403	0.0795
Guizhou	520	108.59	26.52	natural	12900.6085	327545.6389	0.0493
Guizhou	500	109.35	26.63	natural	4362.8510	76025.9179	0.1389
Hunan	410	109.59	26.83	planted	22678.5714	294030.6122	0.0392
Hunan	300	109.75	26.83	natural	5160.0000	57165.7143	0.1750
Hunan	275	109.90	26.90	natural	4544.8276	68279.3103	0.2900
Guizhou	500	109.13	27.03	natural	9081.9209	140706.2147	0.0708
Hunan	615	109.58	27.62	natural	14615.3846	200972.4238	0.0689
Guizhou	1012	107.61	27.70	natural	14362.4161	202662.1924	0.0447
Guizhou	540	108.12	28.25	natural	3264.1509	73193.3962	0.2120
Hunan	500	111.10	28.30	natural	4322.9814	64637.6812	0.2415
Guizhou	1330	106.97	28.47	natural	14425.6121	203258.0038	0.0531
Hubei	680	109.30	29.40	planted	6852.7132	100875.9690	0.1290
Hubei	1100	109.40	30.20	planted	2071.7489	30221.2257	0.3345
Henan	400	114.80	31.60	natural	2418.7500	49925.0000	0.3200
An'hui	400	118.20	32.20	planted	4818.4818	71920.7921	0.1515
Guangxi	10	108.40	21.75	natural	5580.6597	70090.3751	0.2213
Guangdong	250	113.71	23.22	planted	6276.4706	101758.8235	0.1700
Guangdong	200	113.20	23.20	planted	5319.8653	83142.5365	0.1782
Guangxi	450	111.50	24.10	planted	7737.1833	118043.6064	0.1697
Guangxi	520	110.00	25.70	natural	13651.9387	202831.3796	0.1109
Hunan	500	113.30	25.70	natural	4465.3465	67732.6733	0.2020
Fujian	550	117.20	25.80	natural	8617.1063	123461.2310	0.1251
Guangxi	800	110.60	26.00	natural	6722.4670	102757.7093	0.1135
Guangxi	530	110.40	26.00	natural	10971.1846	162881.5368	0.0937
Hunan	483	110.10	26.50	natural	14893.0818	461547.1698	0.0795
Hunan	350	109.60	26.70	natural	9308.8858	134802.5388	0.1418
Hunan	440	109.90	26.80	natural	16047.6788	497804.2660	0.0797
Jiangxi	571	114.70	28.30	natural	9610.8491	235719.3396	0.0848
Jiangxi	217	115.80	29.00	planted	8207.6503	135540.9836	0.0915
An'hui	500	117.90	32.70	planted	3252.7964	42125.2796	0.2235
Guangxi	475	107.65	21.92	natural	2447.9167	39385.4167	0.1920
Guangxi	350	107.07	21.98	planted	2552.9265	41046.0772	0.1606
Guangxi	300	105.08	23.75	planted	1623.1884	92637.6812	0.1725
Guangxi	220	108.68	24.42	planted	4940.3341	310417.6611	0.0838
Guizhou	1420	106.09	26.17	planted	2984.1270	54198.4127	0.1260
Guizhou	1070	108.24	26.27	natural	9820.2614	149852.9412	0.0612

Guizhou	700	108.00	26.33	natural	13235.2941	189936.9748	0.0476
Guizhou	1106	106.73	26.33	planted	3223.2232	58158.1582	0.0999
Guizhou	920	107.83	26.88	natural	7724.2303	123306.5596	0.0747
Guizhou	760	106.60	28.00	natural	3224.7115	58173.7950	0.1473
Guizhou	900	108.11	28.03	natural	4080.9444	71677.9089	0.1186
Zhejiang	500	119.90	28.40	planted	2094.3953	58943.9528	0.1695
Zhejiang	300	119.60	29.10	planted	3205.7971	38602.8986	0.1725
Sichuan	450	106.63	29.72	planted	952.7778	10183.3333	0.3600
An'hui	500	118.15	30.13	planted	2259.6491	76968.4211	0.1425
Hubei	120	112.80	30.80	planted	2769.6477	41121.9512	0.1845
Hubei	650	111.20	31.80	planted	2461.9640	36325.4956	0.2169
Zhejiang	369	120.00	28.50	natural	2546.1648	52070.3125	0.2816
Zhejiang	200	120.60	28.00	planted	5360.6463	66658.9729	0.1733
**Subtropical montane Pinus armandii, P. taiwanensis and P. densada forest**							
An'hui	1700	116.10	31.00	natural	5127.2727	51690.9091	0.2200
An'hui	1050	116.30	30.80	natural	5635.5932	92720.3390	0.2360
Fujian	845	119.30	26.00	natural	11830.9859	299450.7042	0.0710
Fujian	1200	118.90	27.00	natural	5371.4286	118328.5714	0.2100
Fujian	1200	117.10	27.20	natural	3576.0000	52964.0000	0.2500
Gansu	1625	105.88	34.55	natural	1951.4286	18360.0000	0.3500
Guizhou	1850	105.62	27.17	planted	1170.5475	12343.1928	0.4767
Guizhou	1940	104.65	27.05	planted	972.7947	10362.7370	0.3639
Guizhou	1443	104.89	27.13	planted	8745.4324	80621.1937	0.0821
Guizhou	1400	106.82	27.05	planted	10370.9199	114480.7122	0.0674
Guizhou	1680	105.37	26.73	planted	5776.3158	56611.8421	0.1520
Guizhou	1700	105.13	25.68	planted	5545.2436	52720.4176	0.1724
Guizhou	2200	103.84	26.70	natural	3151.6184	71712.0954	0.1174
Guizhou	2360	104.33	26.88	planted	6629.5884	60411.5684	0.0899
Guizhou	2300	104.16	27.02	planted	4277.3723	42948.9051	0.1370
Guizhou	2225	104.20	26.80	natural	8295.4545	190123.9669	0.0968
Guizhou	1920	104.65	24.90	planted	3718.8020	39808.6522	0.1202
Henan	802	115.50	31.50	natural	4139.2801	60352.1127	0.2556
Henan	718	114.80	31.60	natural	6373.0929	89368.9320	0.1442
Hubei	1638	110.30	31.00	planted	4302.0222	34983.6921	0.3066
Hubei	1190	111.70	30.80	natural	5136.8421	85431.5789	0.0950
Hubei	1800	110.60	31.70	natural	5004.6083	71465.4378	0.1085
Hubei	1700	113.60	31.40	planted	1046.0048	10861.9855	0.6195
Hubei	1140	108.90	30.30	natural	21011.9048	392571.4286	0.0840
Hubei	1350	115.70	31.00	planted	2551.2618	34112.7760	0.2536
Hubei	925	113.80	29.20	planted	4825.3968	44463.4921	0.1575
Hubei	120	114.30	29.70	planted	3549.0196	40643.1373	0.2550
Hubei	1500	111.30	30.70	planted	18272.1893	147834.3195	0.0845
Hunan	1433	113.80	28.90	natural	2497.9547	31153.5315	0.3667
Shandong	1000	117.20	36.40	planted	2408.6444	22499.0177	0.2545

Shanxi	1435	105.60	32.67	natural	1204.7619	19352.3810	0.4200
Shanxi	2040	106.60	33.50	natural	5141.3043	122347.8261	0.0920
Shanxi	1787	108.35	33.35	natural	2336.2832	32877.8761	0.2825
Shanxi	1700	107.50	33.20	natural	12437.5000	216156.2500	0.0640
Shanxi	2120	107.20	33.90	natural	3326.9962	81150.1901	0.2104
Sichuan	3445	101.20	27.90	natural	9667.6737	105599.1944	0.0993
Sichuan	3100	101.00	30.00	natural	9085.9799	102680.0930	0.1291
Tibet	2414	95.00	30.10	natural	24340.2778	509270.8333	0.0288
Tibet	2400	94.90	30.20	natural	23387.6221	523745.9283	0.0307
Tibet	2864	95.70	29.80	natural	29774.4361	1171165.4135	0.0266
Tibet	2866	95.70	29.80	natural	25040.6504	680189.7019	0.0369
Tibet	3210	94.10	29.20	natural	21213.0178	370177.5148	0.0338
Tibet	3219	94.10	29.70	natural	12637.7491	206764.3611	0.0853
Tibet	2651	85.20	28.90	natural	16774.1935	303259.7623	0.0589
Tibet	2880	91.90	27.90	natural	6248.8307	78699.7194	0.1069
Tibet	3558	93.10	29.00	natural	20949.3671	423734.1772	0.0316
Tibet	2900	92.30	28.40	natural	20973.7828	363146.0674	0.0267
Tibet	2350	96.70	28.70	natural	28524.5902	1177377.0492	0.0183
Tibet	3000	96.70	28.70	natural	10288.6248	110976.2309	0.1178
Tibet	2900	88.90	27.40	natural	23536.3458	441630.6483	0.0509
Yunnan	3360	99.20	28.25	natural	8066.3507	75251.1848	0.1055
Yunnan	3450	100.11	26.67	natural	3444.4014	20871.7551	0.2581
Yunnan	3152	100.48	27.38	natural	9877.7293	103973.7991	0.1145
Yunnan	3300	99.51	27.53	natural	12095.8084	144481.0379	0.1002
Yunnan	3220	100.96	27.23	natural	2832.7292	15820.8182	0.1931
**Subtropical Cunninghamia lanceolata forest**							
Hainan	780	108.80	18.70	planted	3310.0349	64822.7659	0.2003
Guangxi	500	110.30	21.68	planted	2287.2727	46516.3636	0.2750
Guangxi	460	111.10	23.10	planted	1635.7724	17391.8699	0.3075
Taiwan	1526	120.90	23.70	planted	12939.8254	129871.3826	0.2177
Guangxi	250	109.70	23.80	planted	7688.4058	151065.2174	0.1380
Guangxi	490	110.88	23.88	planted	3524.2930	43226.9761	0.1379
Taiwan	1287	121.12	24.03	planted	14053.8462	194638.4615	0.1300
Guangxi	405	106.23	24.27	planted	2955.3957	22365.4676	0.3475
Fujian	400	117.60	24.40	planted	2454.1667	28875.0000	0.2400
Taiwan	1707	121.10	24.52	planted	16280.4878	124615.8537	0.1640
Guangxi	205	108.75	24.58	planted	3285.0780	39766.1470	0.1796
Fujian	400	117.20	24.70	planted	7650.3067	59325.1534	0.1630
Taiwan	460	121.57	24.83	planted	14176.4706	119529.4118	0.1700
Hunan	624	111.04	25.17	planted	2651.0989	44107.1429	0.2184
Hunan	810	111.12	25.23	planted	2457.8059	40959.9156	0.1896
Guangxi	419	109.09	25.30	planted	4187.5000	53085.9375	0.1280
Guizhou	1445	105.21	25.40	planted	4767.5569	33862.5124	0.2022
Guizhou	461	107.17	25.48	planted	4307.6923	129435.8974	0.1950

Guizhou	365	108.61	25.50	planted	6156.7568	57059.4595	0.1850
Guangxi	966	110.10	25.53	planted	4213.4831	53417.6030	0.1068
Guangxi	750	109.80	25.75	planted	5012.6422	65752.2124	0.1582
Guangxi	380	110.00	25.80	planted	5392.8571	83492.8571	0.1400
Guizhou	430	109.40	26.12	planted	4533.8567	30618.2532	0.2038
Hunan	450	109.50	26.20	planted	5964.9854	97532.3051	0.2399
Jiangxi	220	114.50	26.30	planted	7102.7027	113670.2703	0.1850
Guizhou	1402	106.17	26.48	planted	5840.9091	51164.7727	0.1760
Jiangxi	300	114.40	26.50	planted	5542.6918	87091.1722	0.2073
Guizhou	860	108.91	26.53	planted	6576.8194	65597.4843	0.1113
Fujian	450	118.10	26.60	planted	6472.2222	113152.7778	0.2160
Fujian	450	118.10	26.60	planted	5990.6760	382307.6923	0.1716
Fujian	400	117.90	26.60	planted	8523.7484	164435.1733	0.1558
Guizhou	1400	105.77	26.62	planted	5046.6805	37935.6846	0.1928
Fujian	400	118.20	26.80	planted	7526.1324	138815.3310	0.1435
Guizhou	790	108.99	26.85	planted	5353.0864	42780.2469	0.2025
Guizhou	380	109.43	27.03	planted	6805.2516	70477.0241	0.2285
Guizhou	400	109.35	27.10	planted	4586.7769	31313.1313	0.2178
Guizhou	700	110.23	27.12	natural	2324.2972	38765.0602	0.1992
Guizhou	1010	107.56	27.27	planted	5214.7016	40560.4076	0.2748
Jiangxi	400	114.60	27.30	planted	4673.8072	299795.5209	0.1027
Guizhou	760	108.37	27.53	planted	4188.7338	26186.8079	0.4154
Guizhou	715	108.60	27.82	planted	4799.7284	34324.5078	0.1473
Hunan	220	111.00	28.10	natural	934.1101	15907.9952	0.4978
Guizhou	560	108.92	28.13	planted	5145.2991	39495.7265	0.1170
Guizhou	1427	106.56	28.13	planted	5525.6724	45590.8720	0.1227
Guizhou	660	108.80	28.22	planted	5098.2318	38673.8703	0.1018
Guizhou	712	108.45	28.25	planted	5079.3651	38418.8034	0.1638
Guizhou	1167	105.87	28.35	planted	5666.5181	48100.7579	0.2243
Jiangxi	500	114.50	28.40	natural	1972.2849	28930.0677	0.3103
Guizhou	1060	107.54	28.47	planted	5353.3191	42805.1392	0.1401
Guizhou	1200	107.18	28.65	planted	5101.2536	38717.4542	0.1037
Jiangxi	220	115.72	28.75	planted	7366.0714	73412.6984	0.2016
Guizhou	1100	107.92	28.87	planted	5446.5849	44337.4197	0.1713
Hunan	450	111.13	28.90	planted	3153.5818	36011.1023	0.3783
Hubei	500	113.80	29.20	planted	11639.5864	110302.8065	0.1354
Hubei	1910	109.10	29.60	planted	3396.0047	26180.9636	0.1702
An'hui	450	117.70	29.80	planted	1633.9286	44104.1667	0.3360
Sichuan	1200	103.37	29.92	planted	11670.8229	64324.1895	0.2005
Hubei	600	109.40	30.20	planted	9082.7338	82494.0048	0.1668
Hubei	500	115.70	31.00	planted	6892.1967	59733.8746	0.2217
An'hui	400	116.30	31.30	planted	5211.1111	43083.3333	0.1800
Jiangsu	400	119.80	31.30	planted	4083.3333	32433.3333	0.3000
An'hui	400	115.80	31.60	planted	2437.0370	17829.6296	0.2700

Jiangsu	50	119.51	31.64	planted	3213.8114	24448.8712	0.3765
Henan	200	114.08	32.12	planted	4447.2727	36480.0000	0.2750
Guangxi	400	109.50	22.20	planted	2076.4120	23210.1329	0.2408
Guangdong	200	114.70	22.90	planted	2266.6667	36643.1373	0.2550
Guangdong	400	111.88	23.12	planted	2023.8095	38420.6349	0.2520
Guangxi	400	111.50	24.10	planted	3575.2688	44413.9785	0.1860
Guangxi	720	106.33	24.28	planted	6746.6667	87260.0000	0.1500
Guangdong	400	114.40	24.30	planted	2145.9370	30311.7745	0.3015
Guangxi	1373	106.31	24.40	planted	3984.3750	48246.5278	0.2304
Jiangxi	600	114.50	24.70	planted	6808.3990	100204.7244	0.1905
Jiangxi	600	114.40	24.80	planted	6808.3990	100204.7244	0.1905
Hunan	500	111.50	24.80	natural	3118.5410	51671.7325	0.1645
Hunan	439	111.70	24.90	planted	3326.6833	57715.7107	0.2005
Guangxi	400	109.20	25.00	planted	5853.6585	79750.0000	0.1640
Hunan	343	111.70	25.00	planted	3706.1273	61041.0470	0.1681
Guangdong	500	113.30	25.10	planted	3096.3773	79712.9187	0.1463
Hunan	302	111.30	25.20	planted	2262.0170	37728.5580	0.2122
Fujian	865	116.90	25.68	natural	3281.0458	120882.3529	0.1530
Fujian	400	117.50	26.10	planted	3282.5279	47907.0632	0.2690
Fujian	400	118.10	26.50	planted	6530.1205	115908.4337	0.2075
Guizhou	434	109.10	26.60	planted	7938.3117	104017.8571	0.2464
Hunan	450	109.60	26.70	planted	6468.9936	105577.7702	0.2951
Guizhou	425	109.35	26.75	planted	5468.5176	44645.5131	0.2017
Fujian	403	118.30	26.80	planted	5317.3949	166636.4386	0.1213
Hunan	417	109.50	26.80	planted	4480.7777	216619.5316	0.2263
Fujian	210	117.92	26.80	planted	3270.9187	50295.4944	0.3418
Hunan	400	109.75	26.83	planted	4876.8737	110910.0642	0.1868
Fujian	400	118.60	27.10	planted	4624.2300	73712.5257	0.2435
Fujian	703	117.60	27.50	natural	5808.0808	194507.5758	0.1584
Jiangxi	400	114.60	27.70	planted	2805.3171	28490.7228	0.3611
An'hui	1050	116.20	31.30	planted	5114.0351	46144.7368	0.2280
Shanxi	680	109.30	32.30	natural	6057.4413	59399.4778	0.1532
Jiangsu	20	121.18	32.33	planted	9483.4835	47333.3333	0.1665
Zhejiang	50	120.50	30.48	planted	2433.7349	49429.7189	0.2490
Guangdong	500	112.10	23.90	planted	3200.2706	87868.7415	0.1478
Guangxi	355	109.99	22.55	planted	3725.0294	46351.3514	0.1702
**Subtropical montane Cupressus and Sabina forest**							
Hunan	1235	110.25	25.50	natural	5226.9780	181861.2192	0.1542
Guizhou	1336	106.20	27.40	natural	5982.6087	78602.8986	0.1725
Hunan	200	113.10	27.70	planted	5520.5205	37832.8328	0.1998
Guizhou	780	106.44	28.25	planted	3829.2104	31758.7259	0.2951
Guizhou	1280	105.87	28.28	natural	8227.1062	93267.3993	0.1365
Guizhou	1060	106.48	28.32	natural	9383.0846	112995.0249	0.1005
Sichuan	3100	102.10	28.50	natural	17327.5862	284752.1552	0.0928

Guizhou	500	108.59	28.65	natural	5827.5862	56870.6897	0.1160
Tibet	3445	85.20	28.90	natural	8838.7824	132322.4352	0.0887
Tibet	2300	95.00	30.10	natural	12674.2301	447763.3712	0.0617
Sichuan	3270	102.40	30.90	natural	22234.0426	491303.1915	0.0376
Sichuan	3300	98.80	31.20	natural	7224.3590	82858.9744	0.1560
Sichuan	484	105.15	31.65	planted	6631.0497	38975.4871	0.3182
Sichuan	484	105.18	31.68	planted	9022.2222	60874.0741	0.1350
Sichuan	3500	103.39	33.45	natural	12227.9412	170352.9412	0.1360
Sichuan	3293	103.57	33.59	natural	10077.6197	142147.4774	0.0773

Note: Where the Alt. is altitude, Long. is longitude, Lat. Is latitude, L is leaf mass, M is body mass, and D is density.
