# Supplementary material for: Trade-Offs between the Metabolic Rate and Population Density of Plants
Source: PLoS One. 2008 Mar 19;3(3):e1799. doi: 10.1371/journal.pone.0001799 (PMC2265546; doi:10.1371/journal.pone.0001799)
Supplement: Dataset S2 — The dataset S2 was used in the analysis of our paper (0.13 MB PDF) [file pone.0001799.s002.pdf]

Dataset S2. Desert shrubs data						
For the analysis of metabolic rate			For the analysis of allometric growth			
M(g/no.)	L (g/no.)	D (no./m^2)	D (no./m^2)	M(g/no.)	D (no./m^2)	M(g/no.)
964.2857	100.0000	0.0750	0.0750	950.0000	0. 8889	962. 4087
160.8696	12.1739	0.2700	0.2700	242.0378	18. 0000	57. 7336
185.3333	33.3333	3.3600	3.3600	59.9586	17. 0000	26. 8115
9120.0000	706.6667	3.0000	3.0000	63.7111	10. 0000	4. 0000
600.0000	64.2857	4.0000	4.0000	84.4173	27. 0000	23. 3037
6122.8571	225.7143	0.1667	0.1667	4274.1780	5. 7500	38. 6667
423.0769	70.0000	2.2500	2.2500	152.3465	0. 9089	932. 4087
1318.6567	80.7463	0.1750	0.1750	1314.2857	33. 5239	3. 6908
1100.0000	80.0000	1.3333	1.3333	1012.5000	1. 3000	176. 5188
918.3721	76.7442	0.3600	0.3600	466.7028	0. 0500	1998. 8026
107.6471	8.2353	4.2000	4.2000	44.6698	0. 7375	1464. 2635
702.6667	8.3333	0.3333	0.3333	750.0000	0. 2245	1758. 4035
211.5385	48.1538	2.1111	2.1111	159.0148	0. 1750	964. 2857
483.0000	57.4800	0.6000	0.6000	568.7297	0. 5750	160. 8696
1314.2857	118.5714	0.5889	0.5889	1462.4087	1. 5000	185. 3333
246.0177	22.6549	3.0000	3.0000	59.7147	0. 0750	9120. 0000
4200.0000	236.6667	0.2500	0.2500	4898.2000	1. 5750	600. 0000
1200.0000	50.0000	4.3200	4.3200	152.5788	41. 7864	5. 7872
302.3529	6.0784	7.5000	7.5000	37.7507	0. 3250	423. 0769
700.0000	73.9130	0.8000	0.8000	371.1005	1. 6750	1318. 6567
96.6667	10.2000	2.0000	2.0000	134.5372	0. 2500	1100. 0000
5560.0000	880.0000	0.2500	0.2500	3303.5520	76. 0000	0. 5150
1400.0000	122.5000	12.5000	12.5000	84.5853	0. 4250	107. 6471
575.3398	48.5437	16.2500	16.2500	10.1627	0. 7500	702. 6667
1576.9231	146.1538	0.8333	0.8333	990.0000	0. 3250	211. 5385
460.3333	66.3333	15.2500	15.2500	37.7089	1. 2500	483. 0000
300.0000	26.6667	11.1111	11.1111	26.4765	0. 3600	265. 1438
162.6667	18.0000	33.3333	33.3333	6.2402	2. 8250	246. 0177
1257.1429	128.7143	0.1667	0.1667	1242.0000	27. 6964	26. 3217
380.2500	40.6667	5.2500	5.2500	28.5000	0. 0750	1200. 0000
2666.6667	264.6667	0.7500	0.7500	2686.6667	3. 8250	302. 3529
32.0809	5.7803	1.0000	1.0000	120.3277	0. 5750	700. 0000
950.0000	106.6667	5.5556	5.5556	47.3387	3. 7500	96. 6667
465.2023	41.1561	1.7778	1.7778	300.0000	34. 3100	9. 1721
57.9000	3.9000	4.5000	4. 5000	94. 1842	0. 5000	1400. 0000
141.0000	9.6000	6.2500	6. 2500	61. 0225	2. 5750	575. 3398
31.5000	2.4000	23.5556	23. 5556	13. 8071	0. 3250	1576. 9231
191.6140	44.6000	1.7500	1. 7500	148. 5732	0. 7500	460. 3333
637.0620	93.6150	1.6000	1. 6000	500. 3105	0. 7500	300. 0000
37.7235	4.2300	2.2500	2. 2500	117. 5248	3. 0000	162. 6667
165.0792	13.8840	95.0000	95. 0000	0. 4211	45. 0342	9. 1721

51.7500	4.3500	11.0000	11.0000	20.1729	3.0000	380.2500
76.5300	6.5300	5.0000	5.0000	72.2757	2.7500	57.0000
41.0000	4.5000	220.0000	220.0000	2.6000	4.3250	32.0809
14.0400	2.8860	1.5556	1.5556	121.0000	2.1600	245.6458
120.0000	19.0000	1418.7500	1418.7500	0.8319	4.3250	465.2023
214.5000	30.2025	5.2500	5.2500	32.2500	0.4167	57.9000
926.5665	114.8100	1.0750	1.0750	918.3721	0.0893	181.0000
210.0158	16.4620	102.0000	102.0000	0.3854	0.2500	191.6140
30.7500	1.3500	2.8889	2.8889	50.4000	43.8958	3.4279
60.3878	2.7150	2.1111	2.1111	68.3333	0.5000	37.7235
43.2000	3.0000	64.0000	64.0000	11.5625	0.4167	165.0792
121.6050	11.6300	675.0000	675.0000	0.1019	0.3333	91.7500
2208.0000	291.6000	0.0833	0.0833	2418.0000	0.2500	76.5300
1242.0000	180.0000	0.1750	0.1750	1257.1429	0.5000	41.0000
354.5455	23.4545	213.9908	213.9908	1.3302	0.5000	120.0000
827.6633	66.9675	0.6667	0.6667	1185.4639	0.3933	214.5000
1012.5000	74.2500	0.1667	0.1667	926.5665	72.5095	1.2999
1526.6103	123.4560	0.4167	0.4167	1576.6272	1.2500	210.0158
600.0000	73.6364	13.8811	13.8811	10.7500	1.2500	43.2000
1079.0931	190.6410	13.3482	13.3482	44.9727	0.5000	121.6050
1128.8485	201.2300	19.7435	19.7435	21.3544	16.0000	5.0625
1125.0000	111.0000	16.1619	16.1619	27.4708	51.0000	2.9890
4898.2000	619.4300	0.0750	0.0750	5560.0000	0.9167	354.5455
67.5000	5.5000	201.3500	201.3500	0.7735	0.6667	827.6633
195.0000	17.3775	27.6964	27.6964	20.7029	2.1111	120.5387
750.0000	111.0000	15.9787	15.9787	9.6744	127.0433	0.6111
417.8800	57.4767	96.9224	96.9224	3.1852	0.9167	600.0000
985.7143	85.7143	63.7151	63.7151	5.6275	0.8333	1079.0931
1080.6210	76.6913	0.1667	0.1667	637.0620	0.5000	1128.8485
990.0000	150.0000	73.1547	73.1547	6.1704	0.6667	1125.0000
3303.5520	601.4500	0.0750	0.0750	4200.0000	5.2222	41.0968
1185.4639	188.3025	51.3865	51.3865	4.8718	0.5000	67.5000
7948.8150	1482.8400	0.1750	0.1750	6122.8571	0.3333	195.0000
145.2548	4.4025	10.8498	10.8498	12.4809	2.2222	89.1747
4274.1780	744.4800	0.3889	0.3889	2862.4087	0.7500	417.8800
145.4545	12.2727	17.9284	17.9284	6.4934	0.5833	985.7143
220.0000	19.0000	319.1182	319.1182	0.4063	0.6667	1080.6210
163.3147	25.7486	1464.2722	1464.2722	0.1179	21.2500	12.0348
6.6200	1.6000	2320.7150	2320.7150	0.0477	2.0400	238.0206
28.7900	0.2600	1843.4094	1843.4094	0.1478	218.5327	0.5115
9.2000	0.3600	1605.5425	1605.5425	0.0656	0.0833	7948.8150
17.0000	0.8200	22.0000	22.0000	10.7500	0.8333	145.2548
20.0000	0.6400	576.1853	576.1853	0.0828	5.2500	52.4114
2.8600	0.3900	4531.2500	4531.2500	0.0475	0.5500	145.4545

22.0000	1.0800	9.0000	9.0000	23.4146	0.2500	220.0000
46.8500	2.3000	5.8822	5.8822	44.6156	12.5000	163.3147
5.0000	0.2200	6.2434	6.2434	32.4112	0.5500	145.4545
12.6600	0.6300	587.1030	587.1030	0.1534	0.2500	220.0000
8.6000	0.3000	469.2745	469.2745	0.1894	12.5000	163.3147
28.0000	0.9810	676.3000	676.3000	0.1984		
4.0000	0.1200	1654.6810	1654.6810	0.0361		
24.5000	6.2000	936.3258	936.3258	0.0971		
20.0000	6.3100	851.4113	851.4113	0.2277		
1.2000	0.0800	12.1600	12.1600	48.8821		
46.4600	6.5000	34.3100	34.3100	17.4040		
11.0000	1.0900	201.3500	201.3500	1.2319		
15.0300	2.0000	2859.0255	2859.0255	0.0632		
12.0000	0.2300	17.4690	17.4690	55.1250		
10.9100	0.2100	9.6437	9.6437	82.0052		
7.0000	0.4000	47.3614	47.3614	62.8032		
16.5200	0.9500	743.7500	743.7500	0.4499		
2.4100	0.2700	14.1167	14.1167	9.7800		
42.8300	4.9000	26.0000	26.0000	9.7800		
4.1000	0.2000	676.3000	676.3000	2.7780		
32.8800	1.6800	44.1282	44.1282	0.9289		
25.8000	0.1020	6.2079	6.2079	34.7999		
3.2000	0.0180	1199.9179	1199.9179	0.0302		
35.0000	4.5000	37.0085	37.0085	3.5324		
36.0000	4.6000	48.1963	48.1963	2.7421		
55.1400	22.9900	456.2500	456.2500	0.0438		
65.4700	40.1500	517.9285	517.9285	0.0748		
50.0500	26.7300	17.4380	17.4380	2.7022		
27.9900	14.7600	34.2351	34.2351	9.6830		
24.1100	15.5300	34.2351	34.2351	1.0256		
25.1600	17.2300	17.4380	17.4380	1.6490		
50.2300	29.3700	22.1165	22.1165	5.7730		
80.3900	22.4400	7.8152	7.8152	20.9690		
91.3800	33.7700	1035.5048	1035.5048	0.1541		
58.8600	11.0800	1956.6593	1956.6593	0.0300		
62.8700	29.7000	778.9601	778.9601	0.0963		
86.7500	39.5900	757.0970	757.0970	0.0274		
42.5800	19.2300	67.6300	67.6300	2.7421		
36.7100	13.3600	0.4808	0.4808	365.9299		
56.1700	17.6600	0.3999	0.3999	271.6307		
326.6700	93.4100	583.3380	583.3380	0.0210		
46.8690	19.5415	1284.4413	1284.4413	0.0094		
55.6495	34.1275	6.3437	6.3437	4.4989		
42.5425	22.7205	10.5539	10.5539	4.8207		

23.7915	12.5460	17.1455	17.1455	1.6245
20.4935	13.2005	17.5677	17.5677	12.5029
21.3860	14.6455	28.0000	28.0000	0.9750
42.6955	24.9645	15.4630	15.4630	7.9722
94.8602	26.4792	8.5000	8.5000	36.1209
107.8284	39.8486	618.7500	618.7500	0.0547
69.4548	13.0744	579.4473	579.4473	0.2112
74.1866	35.0460	676.3000	676.3000	0.5780
102.3650	46.7162	0.4900	0.4900	172.4010
50.2444	22.6914	924.5285	924.5285	0.0339
43.3178	15.7648	997.0439	997.0439	0.0125
66.2806	20.8388	26.0000	26.0000	1.6245
385.4706	110.2238	1.5000	1.5000	176.5188
187.8284	69.8486	0.0600	0.0600	2298.8026
202.3650	46.7162	0.4375	0.4375	1634.2635
285.4706	75.2238	0.1224	0.1224	2398.4035
137.8284	19.8486	0.1800	0.1800	459.6718
122.3650	36.7162	0.1400	0.1400	1440.0640
165.4706	65.2238	12.7500	12.7500	62.2318

Note: Where L is leaf mass, M is body mass, and D is density. For the description on sites, see Materials and Methods of the main text.
