# Supplementary material for: Trade-Offs between the Metabolic Rate and Population Density of Plants
Source: PLoS One. 2008 Mar 19;3(3):e1799. doi: 10.1371/journal.pone.0001799 (PMC2265546; doi:10.1371/journal.pone.0001799)
Supplement: Dataset S3 — The dataset S3 was used in the analysis of our paper (0.04 MB PDF) [file pone.0001799.s003.pdf]

Dataset S3. Spring wheat data					
Time	M(g/no.)	L (g/no.)	D (no./m^2)	M(g/no.)	D (no./m^2)
2004	0.4955	0.2237	2001	0.4955	2001
	0.7933	0.1701	1963	0.7933	1963
	1.2003	0.1112	1902	1.2003	1902
	0.1076	0.0324	5985	0.1076	5985
	0.1181	0.0817	5970	0.1181	5970
	0.1535	0.1082	5862	0.1535	5862
	0.3507	0.0700	3217	0.3507	3217
	0.6581	0.0422	2363	0.6581	2363
	0.0328	0.0342	10231	0.0328	10231
	0.0463	0.0335	9925	0.0463	9925
	0.0849	0.0616	9196	0.0849	9196
	0.1489	0.0335	4513	0.1489	4513
	0.1764	0.0242	3842	0.1764	3842
	0.0228	0.0159	16673	0.0228	16673
	0.0346	0.0327	14673	0.0346	14673
	0.0749	0.0634	10031	0.0749	10031
	0.1149	0.0335	7862	0.1149	7862
	0.1336	0.0260	6677	0.1336	6677
	0.0123	0.0083	29469	0.0123	29469
	0.0246	0.0252	24469	0.0246	24469
	0.0463	0.0452	16623	0.0463	16623
	0.0894	0.0260	10424.0710	0.0894	10424.0710
	0.1006	0.0218	6314.2100	0.1006	6314.2100
2003	0.0820	0.0332	2812	0.0820	2812
	0.1670	0.0514	2388	0.1670	2388
	0.4950	0.1113	2480	0.4950	2480
	0.6850	0.0558	2288	0.6850	2288
	0.7810	0.0622	1992	0.7810	1992
	0.0410	0.0210	10112	0.0410	10112
	0.0640	0.0253	9220	0.0640	9220
	0.1300	0.0559	7464	0.1300	7464
	0.2510	0.0400	5424	0.2510	5424
	0.3710	0.0512	2868	0.3710	2868
2002	0.2132	0.0723	1200	0.2132	1200
	0.2759	0.1300	1315	0.2759	1315
	0.4633	0.1805	1315	0.4633	1315
	0.6570	0.1901	1315	0.6570	1315
	1.1688	0.1696	1315	1.1688	1315
	0.0567	0.0339	4720	0.0567	4720
	0.0710	0.0426	4740	0.0710	4740
	0.1425	0.0663	4255	0.1425	4255

	0.2406	0.0696	2915	0.2406	2915
	0.6073	0.0716	2225	0.6073	2225
	0.0316	0.0364	10640	0.0316	10640
	0.0390	0.0246	10267	0.0390	10267
	0.0757	0.0389	9546.6670	0.0757	9546.6670
	0.1319	0.0665	6226.6670	0.1319	6226.6670
	0.1559	0.0466	2945	0.1559	2945

Note: Where L is leaf mass, M is body mass, and D is density.
